# Genetic modulation of brain dynamics in neurodevelopmental disorders: the impact of copy number variations on resting-state EEG

**DOI:** 10.1038/s41398-025-03324-4

**Published:** 2025-04-11

**Authors:** Adrien E. E. Dubois, Elisabeth Audet-Duchesne, Inga Sophia Knoth, Charles-Olivier Martin, Khadije Jizi, Petra Tamer, Nadine Younis, Sébastien Jacquemont, Guillaume Dumas, Sarah Lippé

**Affiliations:** 1 Research Center of the Sainte-Justine Mother and Child University Hospital Center (CHU Sainte-Justine), Montreal, QC H3T 1C5 Canada; 2 Department of Neurosciences, University of Montreal, Montreal, QC H3C 3J7 Canada; 3 Department of Psychology, University of Montreal, Montreal, QC H2V 2S9 Canada; 4 Department of Pediatrics, University de Montreal, Montreal, QC H3T 1C5 Canada; 5 Department of Psychiatry and Addictology, University of Montreal, Montreal, QC H3T 1J4 Canada; 6 Mila – Québec AI Institute, University of Montreal, Montreal, QC Canada

**Keywords:** Clinical genetics, Diagnostic markers

## Abstract

Research has shown that many copy number variations (CNVs) increase the risk of neurodevelopmental disorders (e.g., autism, ADHD, schizophrenia). However, little is known about the effects of CNVs on brain development and function. Resting-state electroencephalography (EEG) is a suitable method to study the disturbances of neuronal functioning in CNVs. We aimed to determine whether there are resting-state EEG signatures that are characteristic of children with pathogenic CNVs. EEG resting-state brain activity of 109 CNV carriers (66 deletion carriers, 43 duplication carriers) aged 3 to 17 years was recorded for 4 minutes. To better account for developmental variations, EEG indices (power spectral density and functional connectivity) were corrected with a normative model estimated from 256 Healthy Brain Network controls. Results showed a decreased exponent of the aperiodic activity and a reduced alpha peak frequency in CNV carriers. Additionally, the study showed altered periodic components and connectivity in several frequency bands. Deletion and duplication carriers exhibited a similar overall pattern of deviations in spectral and connectivity measures, although the significance and effect sizes relative to the control group varied across frequency bands. Deletion and duplication carriers can be differentiated by their periodic power in the gamma band and connectivity in the low alpha band, with duplication carriers showing more disrupted alterations than deletion carriers. The distinctive alterations in spectral patterns were found to be most prominent during adolescence. The results suggest that CNV carriers show electrophysiological alterations compared to neurotypical controls, regardless of the gene dosage effect and their affected genomic region. At the same time, while duplications and deletions share common electrophysiological alterations, each exhibits distinct brain alteration signatures that reflect gene dosage-specific effects.

## Introduction

In the genome, large DNA segments can be deleted (loss of a copy) or duplicated (gain of a copy). As copy number variations (CNVs) are known to be somewhat represented in the genome of healthy individuals and to play an important role in generating variations among the population at large, the severity of their impact on cognition and development varies considerably depending on the genes affected [1]. When specific parts of the genome are affected by CNVs, individuals more frequently exhibit psychiatric disorders, traits, or diseases [2]. Studies have identified several pathogenic CNVs that significantly increase the risk and severity of neurodevelopmental disorders such as autism spectrum disorders (ASD), schizophrenia, and attention deficit hyperactivity disorder (ADHD) [3]. Pathogenic CNVs are identified in 10–15% of children referred to genetic clinics for neurodevelopmental disorders [4].

To date, the vast majority of studies have been conducted on specific CNVs that are recurrent across patient populations. CNVs in the 16p11.2 chromosomal region are among the most prevalent (~3 in 10,000 [5]) and are strongly associated with neurodevelopmental disorders [6]. For instance, deletion (DEL) and duplication (DUP) carriers have a 10-fold increased risk of ASD, and DUP carriers have a 10-fold increased risk of schizophrenia [7, 8]. Twelve recurrent CNVs have been associated individually with ASD [9], eight with schizophrenia [7], and eight with ADHD [10]. Recurrent CNVs have a stable and known impact on the phenotype. However, most pathogenic CNVs identified in patients are rare or nonrecurrent [7, 11, 12]. As they have rarely been identified across patient populations, the statistical power required for individual association studies is difficult to achieve [11]. Studies have shown that rare CNVs are associated with lower IQ [11, 13]. Neurodevelopmental disorders are characterized by significant clinical and etiological heterogeneity, which hinders the reproducibility of research results. The use of biomarkers shared by different genetic etiologies in clinical populations would make it possible to predict a shared developmental risk, independently of psychiatric diagnosis.

How CNVs affect the brain in common pathways that lead to cognitive and behavioral dysfunction is under investigation. While structural MRI studies identified a broad diversity of regional morphometry patterns across genomic loci, decreases in total brain volume and cortical surface area were observed in 20 recurrent CNVs [14], and rare variations are associated with alterations in brain structural asymmetries [15]. CNVs associated with the greatest effects on cognition exhibited the most effect on brain regions [14, 16]. CNVs also distinctively affect brain structure development as age-related trajectories tend to differ between groups [17]. Despite emerging evidence of structural alterations in carriers, very few studies have examined their functional correlates.

Thought to reflect the intrinsic baseline activity of the brain, the resting state (i.e. without task performance) has gained considerable interest in recent years. Alterations in functional connectivity at rest measured by functional magnetic resonance imaging (fMRI) have been shown in 22q11.2 DEL carriers [18, 19]. For instance, widespread dysconnectivity patterns were demonstrated [19]. The gene dosage (i.e. DEL vs. DUP) of recurrent CNVs was shown to be an important factor affecting the brain distinctively. For instance, DEL and DUP at the 16p11.2, 22q11.2, and 1q21.1 loci have been associated with mirror effects on global functional connectivity in fMRI [20, 21].

Electroencephalography (EEG) is well suited to capture the multiple time scales of brain processing, as it records neuronal oscillations on the millisecond scale. The study of resting-state EEG has led to the discovery of brain spectral anomalies and deviant functional organizations in several neurodevelopmental disorders (e.g. [22–28]). Resting-state EEG has then proven to be an effective method to inform us about the delicate dynamics of neuronal functioning found in neuropsychiatric disorders.

EEG studies on specific CNVs have suggested that they affect basic sensory processing. Functional alterations in visual and auditory processing were shown in 22q11.2 DEL carriers compared to controls (CTRL) [19, 29–33]. Furthermore, a few studies on 16p11.2 CNVs revealed distinct response patterns depending on gene dosage. LeBlanc and Nelson (2016) demonstrated greater amplitude visual responses in DEL carriers and reduced amplitude in DUP carriers [34]. Meanwhile, an increased variability in visual brain responses was demonstrated in DEL carriers compared to DUP carriers and controls [35].

Only a few studies have investigated the distinctive EEG resting-state signatures in carriers of recurrent CNVs. It was demonstrated that beta power was significantly higher in 15q11.2-q13.1 DUP carriers compared to ASD and neurotypical children, while delta power was significantly lower [36, 37].

Genetic studies also suggest that CNVs may have critical periods in brain development when their disruptive impact on molecular pathways is greatest. For instance, it appears that genes included in 16p11.2 are expressed early in brain development [38]. Thus, the influence of CNV on phenotype would be expected to appear early and to vary across developmental periods. Sex is an important factor influencing how neurodevelopmental disorders present and develop, but it remains underappreciated in research [39]. It is known that the prevalence of specific neurodevelopmental disorders varies between males and females [40, 41]. It has also been suggested that the male brain requires a milder mutational burden to exhibit neurodevelopmental disorders [42]. Therefore, the effects of sex on CNVs should be further explored regarding electrophysiological brain characteristics.

To date, only one study in MEG investigated the hypothesis of shared connectivity alterations at rest between CNVs at nine different loci [43]. Their findings showed disturbed oscillatory connectivity in the alpha and beta frequency bands in the posterior regions. Thus, we believe that changing gene dosage at any segment of the genome may affect its efficiency and thus alter the development of brain function. A deeper understanding of the shared neural underpinnings within resting-state EEG networks in pathogenic CNV carriers may have strong implications for the evaluation of alterations in neurodevelopmental disorders.

In this study, we aimed to: (1) identify the differences in resting-state EEG signatures in children with pathogenic CNVs compared to neurotypical children; (2) determine the effect of gene dosage on the identified EEG signatures; (3) determine if CNV carriers deviate from the developmental trajectory of the resting-state components; and (4) explore if the identified EEG signatures are modulated by sex. We hypothesized a distinct effect between DEL and DUP carriers on EEG signatures at rest, with larger effect sizes for DEL carriers, and more perturbed resting-state EEG maturational signatures for DEL carriers.

## Materials and methods

### Participants

#### CNV carriers cohort

The data of 109 CNV (66 DEL and 43 DUP) carriers aged from 3 to 17 was collected at Sainte-Justine Mother and Child University Hospital Center (CHU Sainte-Justine, Montreal, Canada). CNV carriers were either probands (*N* = 97), referred to the genetic clinic for the investigation of neurodevelopmental and psychiatric disorders, or siblings of probands (*N* = 12).

#### Neurotypical cohorts

The resting-state EEG raw data of 256 neurotypical controls were obtained from two datasets: the Healthy Brain Network (HBN) dataset [44] for children aged from 5 to 17 years (*N* = 224) and the NED laboratory dataset for children aged 4 years (*N* = 32).

The EEG data for all three cohorts were recorded using a high-density EEG system from Electrical Geodesics, Inc. Table 1 summarizes the demographics of the study participants.

**Table 1:** Participant demographics.

	CNV carriers		Controls	*p*
	Deletions	Duplications		
**N**	66	43	256	
**Age (years)**				
Mean (SD)	6.93 (3.37)	8.12 (3.81)	8.87 (3.41)	0.000*
Range (min-max)	3.02–17.4	3.03–17.0	3.92–17.3	
**Sex (M/F)**	40/26	25/18	140/116	0.662
**CNV inheritance (%)**				
De novo	47.0	41.9	N/A	
Inherited	36.4	41.9	N/A	
Unknown	16.7	16.3	N/A	
**IQ**				
Mean (SD)	77.3 (13.1)	77.5 (13.6)	105.5 (15.9)	0.000*
Range (min-max)	51–106	55–106	70–145	
**Diagnostic (N(%))**				
ASD	17 (25.8)	14 (35.0)	N/A	
ADHD	21 (31.8)	12 (27.9)	N/A	
Learning disorder	7 (10.6)	4 (9.3)	N/A	
ODD	2 (3.0)	0 (0.0)	N/A	
OCD	2 (3.0)	0 (0.0)	N/A	
Impulse-control and conduct disorders	8 (12.1)	1 (2.3)	N/A	
Developmental language disorder (dysphasia)	13 (19.7)	13 (30.2)	N/A	
Developmental coordination disorder (dyspraxia)	7 (10.6)	5 (11.6)	N/A	
Speech-sound disorder	3 (4.5)	4 (9.3)	N/A	
Social communication disorder	1 (1.5)	0 (0.0)	N/A	
Language delay	12 (18.2)	12 (27.9)	N/A	

*IQ* intelligence quotient; *ASD* autism spectrum disorder; *ADHD* attention deficit hyperactivity disorder; *ODD* oppositional defiant disorder; *OCD* obsessive-compulsive disorder.

*p < 0.05.

### Genetic analysis and protocol

Pathogenic CNVs were detected using the genome-wide chromosomal microarray analysis, which is routinely used in several units of the CHU Sainte-Justine. Sixty-nine recurrent and nonrecurrent pathogenic CNVs (50–500 Kb) were identified in this study sample (36 DEL and 33 DUP; 48% and 40% de novo respectively; 59 loci in total). CNVs are detailed in the *SI Appendix*, Table S1. The study protocol was reviewed and approved by the CHU Sainte-Justine Research Ethics Board. All methods were performed in accordance with the relevant guidelines and regulations. All the participants consented to participate in the study and signed the consent form. Eyes-open resting state was recorded for 4 min while participants were watching a movie without sound and subtitles to improve collaboration and reduce motion artifacts through gaze fixation on the screen.

### EEG acquisition

#### CNV and NED cohorts

The testing took place in a dark, soundproof experimental chamber in the CHU Sainte-Justine. Resting-state EEG was recorded using a high-density EEG system with 128 channels (Electrical Geodesics System Inc., Eugene, OR, USA). Signals were acquired and processed by a G4 Macintosh computer using NetStation EEG Software (v. 4.5.4). Impedances were kept below 40 kΩ [45]. Vertex (Cz) was used as a reference. EEG data was digitized at a sampling rate of 1000 Hz and analog bandpass-filtered from 0.1 to 500 Hz.

#### HBN cohort

All EEG data were collected using a 128-channel EEG recording system (Electrical Geodesics System Inc., Eugene, OR, USA). The EEG data were sampled at 500 Hz with a bandpass filter ranging from 0.1 to 100 Hz. The vertex (Cz) was used as the recording reference. Head circumference was measured for each participant to select an appropriately sized EEG net, and electrode impedances were maintained below 40 kΩ.

### EEG signal preprocessing

#### CNV, NED, and HBN cohorts

We developed a Python script using the MNE package (v. 0.23.0) [46] for automatic preprocessing of raw data following these steps: (1) Remove flat channels with a −3 standard deviation threshold; (2) Apply a high-pass filter at 0.1 Hz and a comb filter at 60 Hz (line frequency noise) and its harmonics (120, 180 and 240 Hz); (3) Create a bipolar reference using E8 and E9 that is associated with eye artifacts; (4) Segment continuous EEG signal into 2-second non-overlapping epochs; (5) Remove bad epochs with an adaptive threshold; (6) Re-reference to an average reference; (7) Remove the ICA component associated with eye movements using the bipolar reference; (8) Within each epoch, reject and interpolate channels with remaining artifacts using the Autoreject python library (v.0.3.1) [47]. Twenty-eight electrodes placed around the neck and face prone to muscular artifacts were removed for all participants (see *SI Appendix*, Fig. S1 for locations).

An average of 99/100 (99%) good channels and 109/152 (72%) seconds of clean signals were kept for DEL carriers, 99/100 (91%) channels and 97/152 (64%) seconds for DUP carriers and 99/100 (99%) channels, and 90/132 (68%) seconds for controls.

### EEG signal processing

Further processing was performed in Python using the MNE package (v. 0.23.0) [46].

#### Current source density

Current source density (CSD) epochs based on spherical spline surface Laplacian were computed with 50 iterations (m = 4; λ = 10-5). CSD is a simple and proven mathematical transformation applied to EEG surface potentials that reduces the impact of volume conduction and the reference recording site [48–50].

#### Spectral analysis

Absolute power spectral density (PSD) was estimated using the Welch method [51]. It was applied on 2-second sliding windows, smoothed by a Hamming weighting function, and half-overlapping across epochs (50%). PSD was also log-transformed for normalization and based on median averaging to correct for bias relative to the mean [46]. Instead of using arbitrary frequency bands, the whole spectrum was covered (2.5–45 Hz) with 0.5 Hz frequency bins to better account for inter-subject variability in frequency distribution [52].

The SpecParam algorithm (v. 1.0.0) was then used to parameterize EEG power spectra [53, 54]. Algorithm settings were set as peak width limits: *[1, 8]*; max number of peaks: *6*; minimum peak height: *0.1*; peak threshold: *2*; and aperiodic mode: *fixed*. Power spectra with a poor model fit (i.e., variance explained [R2] < 0.9 or mean absolute error [MAE] > 0.1) were excluded from subsequent analyses (*N* = 12; *SI Appendix*, Fig. S2). The initial fit of the aperiodic slope (1/f-like) was estimated across the whole spectrum and then subtracted from the power spectrum, leaving the power in periodic components [53]. The values of the aperiodic components, specifically the exponents and the offsets, were also extracted.

The frequency peaks were obtained by identifying the frequency within the band (alpha: 2.5–14.5 Hz; beta 15–37 Hz) that exhibits the highest spectral periodic power at an individual level.

#### Functional connectivity analysis

Functional connectivity was estimated using a time-averaged weighted phase lag index (wPLI), a robust method that measures the asymmetry of the distribution of phase differences between two signals [55, 56]. It was computed across the whole spectrum (2.5–45 Hz) and separately for each frequency band of interest: delta (2.5–4 Hz), theta (4–8 Hz), low alpha (8–10 Hz), high alpha (10–12 Hz), low beta (12–20 Hz), high beta (20–30 Hz) and gamma (30–45 Hz). The wPLI was estimated for each epoch across all channels, and then averaged over epochs. A Fisher transform was applied to the wPLI values.

### Normative model

Considering the broad age range of the sample and the non-linear changes in brain measures across childhood and adolescence, spectral and connectivity metrics were adjusted using a normative model estimated on controls with the PyNM package (v. 1.0.0b8; https://github.com/ppsp-team/PyNM; [57]). Normative modeling is an emerging approach that accounts for developmental variations by adjusting measures relative to a neurotypical population [48, 58]. This method has been shown to effectively detect and map distinct patterns of abnormalities in neurodevelopmental disorders such as schizophrenia, ADHD, and autism spectrum disorder (ASD) [48, 59–63].

In this study, the normative model provided normalized measures of the typical developmental trajectory, accounting for non-linear age-related variability. The model was based on Gaussian Process Regression (GPR), which is well-suited for capturing complex, non-linear relationships [64, 65]. For each extracted feature, the average across all valid channels for each participant was computed and used as the dependent variable in the normative model. Outliers were identified using the interquartile range, defined as the range between the 15th percentile and the 85th percentile. Values falling below the 15th percentile minus 1.5 times the interquartile range or above the 85th percentile plus 1.5 times the interquartile range were excluded to reduce the impact of extreme observations.

Age, sex, site, and data quality were included as covariates to predict spectral and connectivity metrics. Site was treated as a categorical variable to account for batch effects, with 0 representing NED and BCAN recordings and 1 representing HBN recordings. Data quality was defined as the ratio of bad epochs identified by Autoreject. The typical developmental trajectory was modeled by training the GPR in the control group. The model’s performance was evaluated using the Standardized Mean Squared Error (SMSE). For additional details, see the PyNM tutorials (https://github.com/ppsp-team/PyNM/).

To assess group differences in spectral and connectivity metrics, one-way ANOVAs were performed on the z-scores obtained from the normative model. Significant univariate results were further analyzed using Tukey post-hoc tests, and effect sizes were quantified using Cohen’s d.

### Regression models

To investigate the relationships between genetic status and gene dosage with age, as well as with sex, regression models were applied to the z-scores.

In *Model 1*, the genetic status (Carriers vs. Non carriers), sex, age and interaction terms were integrated into a general linear model to predict :

$$Y={\beta }_{0}+{\beta }_{1}{Status}+{\beta }_{2}{Sex}+{\beta }_{3}{Age}+{\beta }_{4}{Status}* {Sex}+{\beta }_{5}{Age}* {Status}+\in$$

In order to study gene dosage effect, *Model 2* integrated the gene dosage (DUP vs. DEL vs. Controls), sex, age and interaction terms to predict :

$$\begin{array}{l}Y={\beta }_{0}+{\beta }_{1}{Dosage}+{\beta }_{2}{Sex}+{\beta }_{3}{Age}\\\quad\quad+\,{\beta }_{4}{Dosage}* {Sex}+{\beta }_{5}{Age}* {Dosage}+\in\end{array}$$

## Results

### Characteristics of the population

DEL carriers (6.93 ± 3.37) were significantly younger than DUP carriers (8.12 ± 3.81) and controls (8.87 ± 3.41) (*F*(2, 362) = 8.46, *p* = 0.000, η^2^_*p*_ = 0.04). In each group, there were more male participants than female participants, but the male/female ratio did not significantly differ between DEL, DUP, and controls (X^2^(2) = 0.82, *p* = 0.662; see *SI Appendix*, Fig. S3 for the sex distribution by age in each group). DEL and DUP carriers had significantly lower IQs than controls, which is expected since they are clinical populations (*F*(2, 193) = 74.4, *p* = 0.000, η^2^_*p*_ = 0.435). ASD (28%), TDAH (30%), developmental language disorder (24%), and language delay (22%) were the most prevalent diagnoses among carrier participants. Diagnosis description can be found in Table 1.

### Spectral analysis

#### Power spectral density

The PSD was computed on all electrodes and outliers were removed (delta band: *N* = 3; theta band: *N* = 3; low alpha band: *N* = 3; high alpha band: *N* = 3; low beta band: *N* = 3; high beta band: *N* = 1; gamma band: *N* = 2; whole spectrum: *N* = 3). Subsequently, the normative model was applied.

A one-way ANOVA revealed that carriers showed differences in power normative scores in theta (*F*(2, 362) = 3.16, *p* = 0.044, η^2^_*p*_ = 0.017), high beta (*F*(2, 362) = 6.034, *p* = 0.002, η^2^_*p*_ = 0.032), and gamma (*F*(2, 362) = 11.11, *p* = 0.000, η^2^_*p*_ = 0.058) bands. Table 2 summarizes the results. Post-hoc analyses revealed that both DEL and DUP displayed more power in high beta (DEL-CTRL: *p* = 0.017, *d* = 0.39; DUP-CTRL: *p* = 0.028, *d* = 0.413) and gamma (DEL-CTRL: *p* = 0.000, *d* = 0.532; DUP-CTRL: *p* = 0.001, *d* = 0.571) bands. Additionally, DEL exhibited significantly higher power in the theta band (*p* = 0.036, *d* = 0.344) and the whole spectrum (*p* = 0.01, *d* = 0.415). No significant differences were observed between DUP and DEL. Post-hoc analyses are summarized in Table 3.

**Table 2:** ANOVA analyses of the normative scores between DEL, DUP and neurotypical controls across frequency bands.

Frequency Band	Sum of Squares	df	Mean Square	F	*p*	η²
Power spectral density						
Delta	4.066	2	2.033	2.29	0.103	0.013
Theta	5.924	2	2.962	3.16	0.044*	0.017
Low Alpha	2.085	2	1.042	1.14	0.32	0.006
High Alpha	1.736	2	0.868	0.99	0.372	0.005
Low Beta	3.702	2	1.851	1.938	0.145	0.011
High Beta	12.105	2	6.052	6.034	0.002*	0.032
Gamma	23.492	2	11.746	11.11	0.000*	0.058
Who Spectrum	11.036	2	5.518	5.53	0.004*	0.031
Aperiodic signal						
Exponent	2.286	2	1.143	12.05	0.000*	0.058
Offset	0.199	2	0.099	0.27	0.766	0.002
Periodic signal						
Delta	15.329	2	7.665	6.35	0.002*	0.035
Theta	21.696	2	10.848	8.7	0.000*	0.047
Low Alpha	13.913	2	6.957	7.25	0.001*	0.039
High Alpha	16.429	2	8.214	8.48	0.000*	0.046
Low Beta	6.437	2	3.218	2.85	0.059	0.016
High Beta	11.716	2	5.858	6.08	0.003*	0.033
Gamma	11.654	2	5.827	6.44	0.002*	0.036
Whole Spectrum	15.887	2	7.943	6.4	0.002*	0.037
Frequency peak						
Alpha	34.672	2	17.336	18.63	0.000*	0.097
Beta	7.809	2	3.904	3.89	0.021*	0.022
Connectivity						
Delta	0.304	2	0.152	0.19	0.831	0.001
Theta	14.247	2	7.124	7.7	0.001*	0.042
Low Alpha	13.546	2	6.773	7.66	0.001*	0.041
High Alpha	9.3	2	4.65	4.81	0.009*	0.026
Low Beta	8.074	2	4.037	4.44	0.012*	0.025
High Beta	6.127	2	3.063	3.58	0.029*	0.02
Gamma	3.203	2	1.602	1.87	0.156	0.011
Whole Spectrum	7.575	2	3.788	4.04	0.018*	0.022

*p < 0.05.

**Table 3:** Post-hoc analyses of the normative scores between DEL, DUP and neurotypical controls across frequency bands.

Frequency Band	Comparison	Mean Difference	*p*	Lower CI	Upper CI	Cohen’s d
Power Spectral Density						
Delta	DEL - CTRL	0.276	0.087	−0.030	0.582	0.297
	DUP - CTRL	0.012	0.996	−0.353	0.378	0.013
	DEL - DUP	−0.263	0.327	−0.698	0.170	−0.289
Theta	DEL - CTRL	0.331	0.036*	0.015	0.646	0.344
	DUP - CTRL	−0.002	0.999	−0.378	0.373	−0.002
	DEL - DUP	−0.333	0.186	−0.78	0.113	−0.372
Low Alpha	DEL - CTRL	0.086	0.791	−0.224	0.396	0.090
	DUP - CTRL	−0.193	0.435	−0.564	0.176	−0.198
	DEL - DUP	−0.279	0.294	−0.720	0.160	−0.314
High Alpha	DEL - CTRL	−0.026	0.977	−0.330	0.278	−0.028
	DUP - CTRL	−0.217	0.338	−0.580	0.146	−0.225
	DEL - DUP	−0.190	0.551	−0.622	0.24	−0.214
Low Beta	DEL - CTRL	0.251	0.151	−0.066	0.569	0.262
	DUP - CTRL	0.154	0.604	−0.225	0.533	0.152
	DEL - DUP	−0.097	0.867	−0.547	0.353	−0.105
High Beta	DEL - CTRL	0.379	0.017*	0.054	0.705	0.390
	DUP - CTRL	0.424	0.028*	0.035	0.813	0.413
	DEL - DUP	0.044	0.971	−0.417	0.506	0.044
Gamma	DEL - CTRL	0.526	0.000*	0.192	0.861	0.532
	DUP - CTRL	0.594	0.001*	0.195	0.993	0.571
	DEL - DUP	0.067	0.939	−0.406	0.542	0.062
Whole Spectrum	DEL - CTRL	0.403	0.010*	0.078	0.728	0.415
	DUP - CTRL	0.341	0.097	−0.046	0.728	0.332
	DEL - DUP	−0.062	0.945	−0.522	0.398	−0.062
Aperiodic Signal						
Exponent	DEL - CTRL	−0.567	0.000*	−0.929	−0.205	−0.527
	DUP - CTRL	−0.44	0.048*	−0.877	−0.002	−0.428
	DEL - DUP	0.127	0.831	−0.390	0.645	0.094
Offset	DEL - CTRL	−0.033	0.969	−0.362	0.295	−0.032
	DUP - CTRL	0.100	0.823	−0.297	0.498	0.108
	DEL - DUP	0.013	0.781	−0.336	0.604	0.119
Periodic Signal						
Delta	DEL - CTRL	0.552	0.001*	0.187	0.917	0.521
	DUP - CTRL	0.119	0.794	−0.316	0.555	0.115
	DEL - DUP	−0.432	0.123	−0.951	0.086	−0.321
Theta	DEL - CTRL	0.624	0.000*	0.255	0.992	0.582
	DUP - CTRL	0.355	0.144	−0.088	0.798	0.340
	DEL - DUP	−0.269	0.450	−0.795	0.256	−0.192
Low Alpha	DEL - CTRL	−0.514	0.000*	−0.837	−0.191	−0.536
	DUP - CTRL	−0.213	0.391	−0.598	0.170	−0.212
	DEL - DUP	0.300	0.271	−0.157	0.758	0.314
High Alpha	DEL - CTRL	−0.517	0.000*	−0.841	−0.193	−0.549
	DUP - CTRL	−0.381	0.054	−0.767	0.005	−0.371
	DEL - DUP	0.136	0.765	−0.323	0.596	0.138
Low Beta	DEL - CTRL	−0.307	0.099	−0.657	0.043	−0.300
	DUP - CTRL	−0.272	0.275	−0.689	0.145	−0.265
	DEL - DUP	0.035	0.985	−0.462	0.532	0.028
High Beta	DEL - CTRL	−0.138	0.572	−0.462	0.185	−0.14
	DUP - CTRL	−0.566	0.001*	−0.952	−0.181	−0.588
	DEL - DUP	−0.428	0.073	−0.887	0.030	−0.423
Gamma	DEL - CTRL	0.045	0.940	−0.273	0.364	0.047
	DUP - CTRL	−0.556	0.001*	−0.933	−0.178	−0.58
	DEL - DUP	−0.601	0.005*	−1.052	−0.150	−0.664
Whole Spectrum	DEL - CTRL	−0.240	0.279	−0.611	0.130	−0.228
	DUP - CTRL	−0.642	0.001*	−1.080	−0.204	−0.608
	DEL - DUP	−0.401	0.167	−0.924	0.120	−0.286
Frequency Peak						
Alpha	DEL - CTRL	−0.806	0.001*	−1.131	−0.482	−0.882
	DUP - CTRL	−0.442	0.017*	−0.821	−0.063	−0.485
	DEL - DUP	0.364	0.145	−0.091	0.819	0.299
Beta	DEL - CTRL	0.399	0.015*	0.062	0.737	0.399
	DUP - CTRL	0.067	0.915	−0.326	0.461	0.069
	DEL - DUP	−0.333	0.223	−0.805	0.140	−0.311
Connectivity						
Delta	DEL - CTRL	−0.056	0.895	−0.353	0.240	−0.061
	DUP - CTRL	0.049	0.942	−0.305	0.405	0.052
	DEL - DUP	0.106	0.824	−0.316	0.528	0.158
Theta	DEL - CTRL	0.504	0.000*	0.189	0.820	0.522
	DUP - CTRL	0.281	0.199	−0.104	0.667	0.289
	DEL - DUP	−0.223	0.481	−0.678	0.232	−0.247
Low Alpha	DEL - CTRL	0.020	0.988	−0.300	0.340	0.020
	DUP - CTRL	−0.501	0.007*	−0.891	−0.111	−0.503
	DEL - DUP	−0.521	0.021*	−0.981	−0.061	−0.55
High Alpha	DEL - CTRL	−0.329	0.031*	−0.635	−0.024	−0.345
	DUP - CTRL	−0.532	0.002*	−0.905	−0.160	−0.55
	DEL - DUP	−0.203	0.525	−0.643	0.237	−0.253
Low Beta	DEL - CTRL	−0.190	0.330	−0.504	0.124	−0.193
	DUP - CTRL	−0.458	0.014*	−0.840	−0.076	−0.48
	DEL - DUP	−0.267	0.345	−0.720	0.184	−0.315
High Beta	DEL - CTRL	−0.273	0.089	−0.578	0.032	−0.291
	DUP - CTRL	−0.308	0.119	−0.675	0.058	−0.318
	DEL - DUP	−0.035	0.980	−0.470	0.400	−0.048
Gamma	DEL - CTRL	−0.241	0.153	−0.546	0.064	−0.253
	DUP - CTRL	−0.132	0.673	−0.499	0.235	−0.138
	DEL - DUP	0.108	0.827	−0.327	0.544	0.150
Whole Spectrum	DEL - CTRL	0.368	0.018*	0.050	0.685	0.382
	DUP - CTRL	−0.052	0.946	−0.436	0.332	−0.053
	DEL - DUP	−0.419	0.077	−0.874	0.035	−0.449

*p < 0.05.

#### Aperiodic signal

**Fig. 1:**
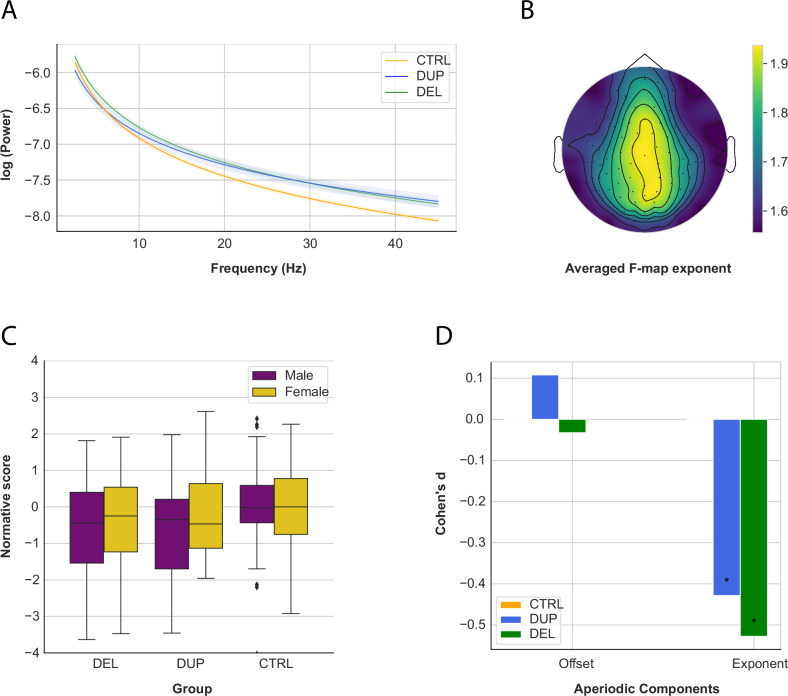
Results of the aperiodic signal. **A** Aperiodic power spectra (in log) at rest from 2.5 to 45 Hz. **B** Average F-map of the exponent. **C** Normative score of the exponent by sex in DEL, DUP and CTRL. Male carriers exhibited significantly lower power compared to females (*B* = −0.774, *p* = 0.047). **D** Effect sizes of the aperiodic components in DEL, DUP and CTRL. * *p* < 0.05.

The average exponent and offset were computed across all electrodes (Fig. 1A, B), and participant outliers were removed (exponent: *N* = 3; offset: *N* = 4). Subsequently, the normative model was applied (*SI Appendix*, Fig. S5 (top)).

Significant group differences were observed in the exponent normative scores (*F*(2, 362) = 12.05, *p* = 0.000, η^2^_*p*_ = 0.058), while no significant differences were observed for the offset (Fig. 1D and Table 2). Post-hoc analyses showed significantly smaller exponents in DEL (*p* = 0.000, *d* = −0.527) and DUP (*p* = 0.048, *d* = −0.428) compared to CTRL, with no significant differences between DEL and DUP (Table 3).

We applied regression models to predict the aperiodic (offset and exponent) normative scores based on two approaches: (1) genetic status (carriers vs. controls), sex, and age; and (2) gene dosage (DEL vs. DUP vs. controls), sex, and age (see *SI Appendix*, Table S2). The interaction of Age * Genetic status significantly predicted the offset (Model 1: *B* = − 0.082, *p* = 0.014), with older carriers exhibiting smaller offset. The interaction of Sex * Genetic status was also significant (Model 1: *B* = 0.780, *p* = 0.001). Male carriers, particularly DEL, were found to have a lower offset (Model 2: DEL, *B* = − 0.886, *p* = 0.002; DUP, *B* = − 0.663, *p* = 0.055). Regarding the exponent, the interaction of Sex * Gene dosage showed that male DUP carriers exhibited a smaller exponent compared to controls (Model 1: *B* = − 0.774, *p* = 0.047). Age-normative trajectories of significant interactions are shown in *SI Appendix*, Fig. S4.

#### Periodic signal

**Fig. 2:**
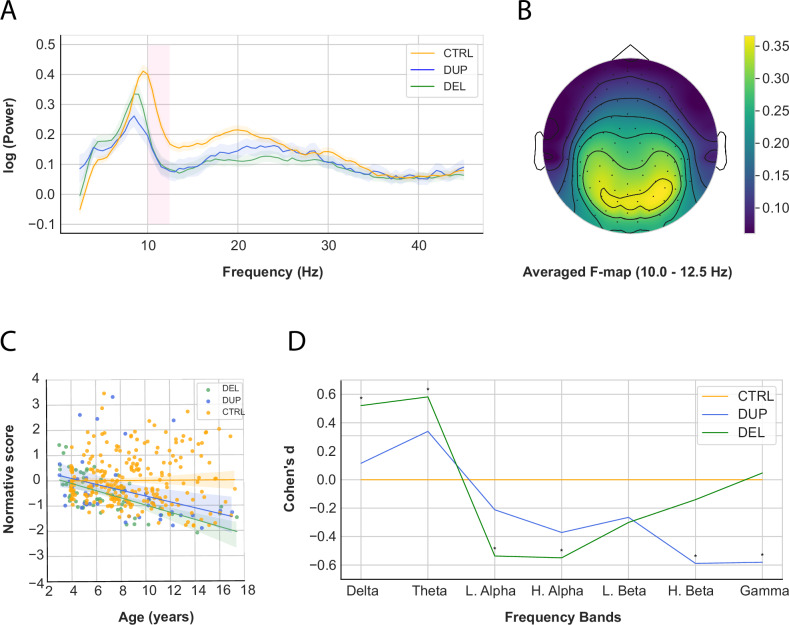
Results of the periodic signal. **A** Periodic power spectra (in log) at rest from 2.5 to 45 Hz. **B** Average F-map of the periodic high alpha signal. **C** Developmental trajectory of periodic power in the high alpha band for DEL, DUP, and CTRL groups. Note that the power is now a normative score after applying the normative model. Shaded regions represent the standard error. Older CNV carriers showed less power (DEL: *B* = −0.144, *p* = 0.000; DUP, *B* = −0.113, *p* = 0.008) **D** Effect sizes of periodic normative scores between DEL, DUP, and CTRL across frequency bands. * *p* < 0.05.

The average periodic power was computed across all electrodes for each frequency band (Fig. 2A, B) and participant outliers were removed (delta band, *N* = 4; theta band, *N* = 3; low alpha band, *N* = 0; high alpha band, *N* = 1; low beta band, *N* = 0; high beta band, *N* = 2; gamma band, *N* = 8; whole spectrum, *N* = 8). Subsequently, the normative model was applied (*SI Appendix*, Fig. S6).

Significant group differences in periodic power normative scores were observed across all frequency bands except low beta (Table 2). DEL and DUP showed a similar pattern on average, with increased power in lower frequencies and decreased power in higher frequencies (Fig. 2D). However, DEL showed significant differences in lower frequencies and DUP in higher frequencies. DEL exhibited more power in delta (*p* = 0.001, *d* = 0.521) and theta (*p* = 0.000, *d* = 0.582) bands, but less in low alpha (*p* = 0.000, *d* = −0.536) and high alpha (*p* = 0.000, *d* = −0.549) bands. DUP showed less power in high beta (*p* = 0.001, *d* = −0.588), gamma (*p* = 0.001, *d* = −0.580) bands and the whole spectrum (*p* = 0.001, *d* = −0.608), with a similar trend in the high alpha band (*p* = 0.054, *d* = −0.371). Finally, DUP exhibited less power than DEL in the gamma band (*p* = 0.005, *d* = −0.664) (Table 3).

We then applied the regression models predicting the periodic normative score (*SI Appendix*, Table S3). The interaction term Age * Genetic status was a significant predictor of the periodic power normative score for several frequency bands. Older CNV carriers showed more periodic power in theta (*B* = −0.129, *p* = 0.000), low alpha (*B* = −*0.079, p* = 0.012) and gamma (*B* = *−0.064, p* = 0.046) bands, but less in high alpha (*B* = *0.124, p* = 0.000) and low beta (*B* = *0.115, p* = 0.000) bands (Model 1) (see Fig. 2C and SI *Appendix* Fig. S4). Older DEL and DUP showed more periodic power than controls in the theta band (DEL: *B* = 0.110, *p* = 0.014, DUP: *B* = 0.166, *p* = 0.000), while for the high alpha band they showed less power (DEL: *B* = −0.144, *p* = 0.000; DUP, *B* = −0.113, *p* = 0.008) (Model 2).

#### Frequency peaks

The average frequency peaks were computed across electrodes, and no outliers were detected (alpha peak: *N* = 0; beta peak: *N* = 0). Subsequently, the normative model was applied (*SI Appendix*, Fig. S5 (bottom)).

Significant group differences were observed in alpha (*F*(2, 362) = 18.63, *p* = 0.000,η^2^_*p*_ = 0.097) and beta (*F*(2, 362) = 3.89, *p* = 0.021, η^2^_*p*_ = 0.022) peaks (Table 2). Post-hoc analyses showed that DEL (*p* = 0.001, *d* = −0.882) and DUP (*p* = 0.017, *d* = −0.485) exhibited reduced alpha frequency peaks compared to CTRL. DEL also showed a significant increase in the beta peak compared to CTRL (*p* = 0.015, *d* = 0.399) (Table 3).

No significant interaction terms were observed for frequency peaks. (SI Appendix, Table S4).

### Connectivity

**Fig. 3:**
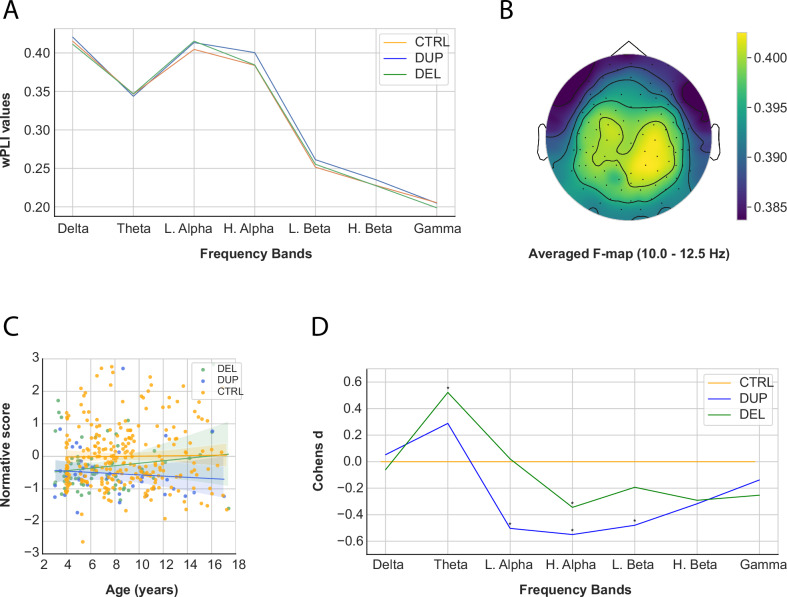
Results of the connectivity signal. **A** Average connectivity across frequency bands. **B** Average F-map of connectivity in the high alpha band. **C** wPLI developmental trajectory in the high alpha band in carriers (DEL and DUP) and neurotypical controls (CTRL). Shaded regions represent the standard error. No significant differences were observed between groups. **D** Effect sizes of normative scores between DEL, DUP, and CTRL across frequency bands. *p < 0.05.

The average wPLI of all electrodes was computed for each frequency band to identify and exclude participant outliers (delta band: *N* = 4; theta band: *N* = 11; low alpha band: *N* = 5; high alpha band: *N* = 3; low beta band: *N* = 11; high beta band: *N* = 9; gamma band: *N* = 11; whole spectrum: *N* = 8) (Fig. 3A, B). Subsequently, the normative model was applied (*SI Appendix*, Fig. S7).

The wPLI normative scores revealed significant group differences in theta (*F*(2, 362) = 7.70, *p* = 0.001, η^2^_*p*_ = 0.042), low alpha (*F*(2, 362) = 7.66, *p* = 0.001, η^2^_*p*_ = 0.041), high alpha (*F*(2, 362) = 4.81, *p* = 0.009, η^2^_*p*_ = 0.026), low beta (*F*(2, 362) = 4.44, *p* = 0.012, η^2^_*p*_ = 0.025), high beta (*F*(2, 362) = 3.58, *p* = 0.029, η^2^_*p*_ = 0.020) bands and the whole spectrum (*F*(2, 362) = 4.04, *p* = 0.018, η^2^_*p*_ = 0.022) (Table 2). Post-hoc analyses indicated that both DEL and DUP groups had lower wPLI normative scores in the high alpha band compared to CTRL (DEL-CTRL: *p* = 0.031, *d* = −0.345; DUP-CTRL: *p* = 0.002, *d* = −0.550). DUP exhibited reduced connectivity compared to CTRL in low alpha (*p* = 0.007, *d* = −0.503) and low beta bands (*p* = 0.014, *d* = −0.480). In contrast, DEL exhibited increased connectivity compared to CTRL in the theta band (*p* = 0.000, *d* = 0.522) and the whole spectrum (*p* = 0.018, *d* = 0.382). DUP showed significantly reduced connectivity compared to DEL in the low alpha band (*p* = 0.021, *d* = −0.550) (Fig. 3D and Table 3).

No significant interaction terms were observed for connectivity (Fig. 3C, *SI Appendix*, Table S5).

## Discussion

Our goal was to determine resting-state EEG signatures, in a well-powered cohort of children carriers of pathogenic CNVs, to examine the effect of gene dosage (DEL vs. DUP) on functional brain anomalies. Furthermore, we investigated how the brain signals of individuals with pathogenic CNVs evolve through development and how they are modulated by sex. The spectral density of carriers followed a significant increase in higher frequencies. These results are consistent with several reports of spectral density patterns in NDD [36, 66]. Nevertheless, our study highlights the importance of conducting SpecParam analyses in conjunction with PSD. While the aperiodic signal analyses align with the power spectrum results showing a lower exponent in CNV carriers, the periodic signal analyses revealed a gene dosage effect. More precisely, DEL carriers show increased lower frequency periodic signals, while DUP carriers show decreased higher frequency periodic signals. Both groups showed reduced alpha periodic signals. Age was a significant predictor of periodic activity with older CNV carriers presenting the more severe electrophysiological phenotype in theta, high alpha and low beta bands. Whether the age effect is due to aggravated pathophysiology in CNV carriers over time or if it is the result of neurodevelopmental stagnation needs to be investigated through longitudinal studies. Brain connectivity alterations in carriers were found on the whole spectrum as well as most frequency bands. Notably, while CNV carriers generally exhibited hypoconnectivity in the alpha band, DEL carriers showed hyperconnectivity in delta, and DUP carriers demonstrated hypoconnectivity in low alpha and low beta. Finally, both groups showed reduced alpha peak frequency. Taken together, our findings demonstrate resting-state EEG alterations shared between different recurrent and rare pathogenic CNVs associated with neurodevelopmental disorders. These are in line with emerging literature proposing common neurobiological substrates for mental disorders [20, 67]. Convergent neural alterations may help clarify what mechanisms link CNVs at different loci to a shared disorder. Gene dosage effects can be found in some specific electrophysiological features.

### Spectral characteristics of CNV carriers

Our results indicate that CNV carriers display a significant alteration in PSD, namely an increase in high beta and gamma bands. Additionally, DEL exhibited more power in the theta band. These findings align with prior studies, such as Frohlich et al. (2016) and Dangles et al. (2022), which reported higher beta power at rest in children carrying the 15q11.2-q13.1 DUP [36, 66]. Saravanapandian et al. (2020) stated that spontaneous and large amplitude beta oscillations are an EEG biomarker for carriers in the 15q syndrome [37]. Similar findings have been reported in 16p11.2 DEL carriers [68]. Atypical beta and gamma power have also been observed in several neurodevelopmental disorders, including ASD [69–71], Fragile X Syndrome [26, 72–75], and are considered markers of schizophrenia [76–78]. Similarly, SYNGAP1 mutations, a rare condition associated with intellectual disability and ASD, have been linked to increased beta and gamma power [79, 80].

The increase in high beta and gamma bands is likely associated with the aperiodic component. The exponent showed an important reduction among CNV carriers, driving the increased broadband power in higher frequencies.

Regarding the periodic signal, DEL and DUP exhibited similar patterns on average, characterized by increased power in low-frequency bands (delta and theta) and reduced power in the alpha range. However, their deviations are not significant in the same frequency bands. DEL showed stronger deviations, with significant increases in delta and theta power and greater reductions in the alpha range, while DUP displayed more pronounced reductions in high beta and gamma bands on the periodic signal. The gamma band is the only frequency range where DEL and DUP differed significantly. Additionally, the alpha peak frequency is significantly reduced among CNV carriers, a hallmark of several neurodevelopmental disorders [26, 75]. Also, DEL showed an increased beta peak frequency. Beta peak frequency has been associated with both neurological statuses and behavioral measures in other CNVs [37]. Our results align with beta peak frequency being an emerging relevant EEG biomarker.

Hence, the difference in spectral characteristics between periodic (rhythmic) and aperiodic (non-rhythmic) activity in CNV carriers may reflect impairments in brain circuits responsible for generating oscillations typical of organized processing, resulting in both more chaotic non-rhythmic activity and disorganized rhythmic activity. As previously posited, enhanced background activity, or network noise, may interfere with the generation of organized rhythmic activity crucial to information processing. Notably, gamma oscillations—critical for maximal information processing—reflect synchronized neuronal activity [81–83]. At a pathophysiological level, a large portion of the CNVs included in this study may affect GABAergic neurons [84]. In particular, parvalbumin-expressing (PV) cells are required to generate and maintain gamma oscillations and their alterations have been shown to contribute to increased baseline cortical gamma rhythm [85–89]. Hence, our results support the need for studies investigating specific GABAergic cell subgroups in CNVs.

### Connectivity characteristics of CNV carriers

Both DEL and DUP exhibit a similar trend in average of increased connectivity in theta and reduced connectivity from alpha to higher frequencies. However, these patterns are only significant for both groups in the high alpha band. Additionally, DEL showed significantly more connectivity in the theta band and the whole spectrum while DUP exhibited significantly less connectivity in low alpha and low beta bands. The low alpha band is the only frequency range where they differ significantly, with DUP exhibiting less connectivity than DEL.

Atypical connectivity signatures appear to be generalizable, as they show convergence across various CNVs and diverse clinical phenotypes. Our findings replicate those of Dima et al. (2020) on 14 recurrent and rare CNVs which found alpha and beta bands oscillatory dysconnectivity in carriers [43]. The deficits in information integration at the neural and cognitive levels found in neurodevelopmental disorders may be the result of under-functioning neural circuitry caused by the CNVs (under-connectivity theory [90, 91, 92]). Our results are also in agreement with Moreau et al. (2020, 2021) studies on 16p11.2, 22q11.2, 1q21.1, 15q11.2, and 2q13 CNVs. Like their findings, we observed alterations in global connectivity, regardless of the genomic location of the CNVs [20, 21]. Our study is also consistent with previous studies showing various resting-state architectures of altered connectivity in several neurodevelopmental disorders [69, 93, 94].

### Gene dosage effect

We aimed to determine whether DEL and DUP carriers exhibit distinct atypical resting-state EEG signals, potentially reflecting differential gene-dosage effects on neurodevelopment. While both groups displayed a similar overall pattern of deviations in spectral and connectivity measures, the significance and effect sizes relative to the control group varied across frequency bands. DEL and DUP carriers could only be differentiated from each other in the periodic gamma band and low alpha connectivity, with DUP showing significantly less power in gamma and reduced connectivity in low alpha compared to DEL, seemingly demonstrating a more severe phenotype. The clinical context of our sample may have influenced these findings, as the DUP group comprises individuals with more severe impairments compared to the general population. Additionally, the heterogeneity in CNVs within our sample may have mitigated some effects within both DEL and DUP groups.

Nevertheless, previous studies have documented opposing effects of DEL and DUP on body weight, head circumference, brain volume, and basic sensory processing [34, 95–98]. Our findings provide additional nuance to this understanding, revealing a shared global pattern for DEL and DUP, with differences in the amplitude of their deviations across frequency bands. Previous evidence further suggests that pathogenic DEL tends to have more severe consequences on phenotype and brain function compared to DUP [95, 99]. Here, in the two cases where DEL and DUP directly differ in our study—periodic gamma power and low alpha connectivity—DUP exhibited stronger deviations.

The distinct patterns between DEL and DUP carriers may provide valuable insights into differences in brain organization associated with these genetic conditions and highlight the potential of EEG as a tool for differentiation. Our study highlights the importance of nuancing results according to recruitment biases.

### Resting-state EEG developmental trajectories in CNV carriers

Our study provided an investigation of the age-related effects of pathogenic DEL and DUP on brain function between the ages of 3 and 17 years. Our analyses revealed aperiodic (offset) and periodic alterations (particularly in theta, low alpha, high alpha, low beta and gamma bands) with age in CNV carriers, suggesting a divergent developmental trajectory in resting-state activity. Mancini et al. (2022) found a divergent developmental trajectory in power and connectivity [33]. It has been proposed that disturbances in adolescent brain maturation may play a significant role in the pathophysiology of schizophrenia [100]. In our sample, the alterations were most prominent at older ages for the offset and theta, high alpha, and low beta bands, which correspond to the onset period of schizophrenia symptomatology. Several CNVs have been found to potentially contribute to the onset of schizophrenia, including several loci present in our sample (i.e., 1q21.1, 16p11.2, 22q11.2) [7]. However, in this study, no relative contribution from a specific CNV can account for the results, and no participant presented with a schizophrenia diagnosis. Overall, this study’s results point to potential developmental impairments in the circuitry underlying the maturation of neural oscillations in CNV carriers during adolescence. Furthermore, it would be relevant to investigate if these age-related changes in resting-state connectivity persist into adulthood.

### Sex modulation

We aimed to explore the modulation effect of sex on the EEG signatures at rest. Our results suggest that male DEL carriers exhibit a reduced offset, while male DUP carriers show a reduced exponent on the aperiodic signal compared to female carriers. An increased prevalence of neurodevelopmental disorders is observed in males [101]. Existing literature also showed that males are more likely to be referred for genetic testing than females carrying the same CNV. Studies on the 16p11.2 CNVs have reported an increase in the frequency of males for both DEL and DUP [96, 102]. Our findings thus raise the hypothesis that a disruption of the neural circuitry in male CNV carriers may contribute to the overrepresentation of males in neurodevelopmental disorders. However, additional evidence would be necessary to confirm this hypothesis.

### Limitations

We must acknowledge some limitations of our study. We have decided to perform an eyes-open resting-state, since many individuals with neurodevelopmental disorders are not able to perform the eyes-closed condition. However, it has been brought up that the eyes-open resting state condition is highly variable as visual input and attention are likely to vary across subjects during recording and could influence our overall results due to differences in visual processing [34], suggesting eyes-closed as a more uniform condition [24]. Therefore, it would be interesting to compare the two resting-state conditions in further research. We are also reporting cross-sectional data, so it would be pertinent to confirm our results on developmental trajectories by longitudinal studies. Although variability within CNV carriers may have obscured specific effects, the detection of group effects indicates robust EEG alterations across distinct genotypes and phenotypes.

## Conclusion

In this study, we demonstrated that CNV carriers present resting-state EEG alterations at the spectral and connectivity levels. We identified consistent disruptions in power and connectivity, with some distinct patterns linked to gene dosage effects. DEL carriers displayed increased power in low-frequency bands and hyperconnectivity in specific ranges, while DUP carriers exhibited reduced power and hypoconnectivity, particularly in higher frequencies. These findings underline the potential of electrophysiological biomarkers in differentiating the neurobiological effects in genetic clinical conditions. Adolescence appears to be a critical stage of development during which mutation-induced effects from CNVs are seemingly most detrimental to brain oscillations and connectivity. This is the first EEG study that offers generalizable spectral and connectivity signatures for multiple pathogenic CNVs, both rare and recurrent. Our study adds to a growing understanding of how pathogenic CNVs disrupt brain homeostasis in ways that lead to the neuropsychological profiles observed in clinical disorders. It also opens the possibility of electrophysiological biomarkers shared across multiple pathogenic CNVs. Future studies should investigate whether the observed effects can be linked to molecular-level mechanisms.

## Supplementary information

Supplementary material

## Data Availability

HBN data is publicly available. BCAN and NED data are available from the corresponding author upon request.
