# Supplementary material for: Genetic modulation of brain dynamics in neurodevelopmental disorders: the impact of copy number variations on resting-state EEG
Source: Transl Psychiatry. 2025 Apr 11;15:139. doi: 10.1038/s41398-025-03324-4 (PMC11992136; doi:10.1038/s41398-025-03324-4)

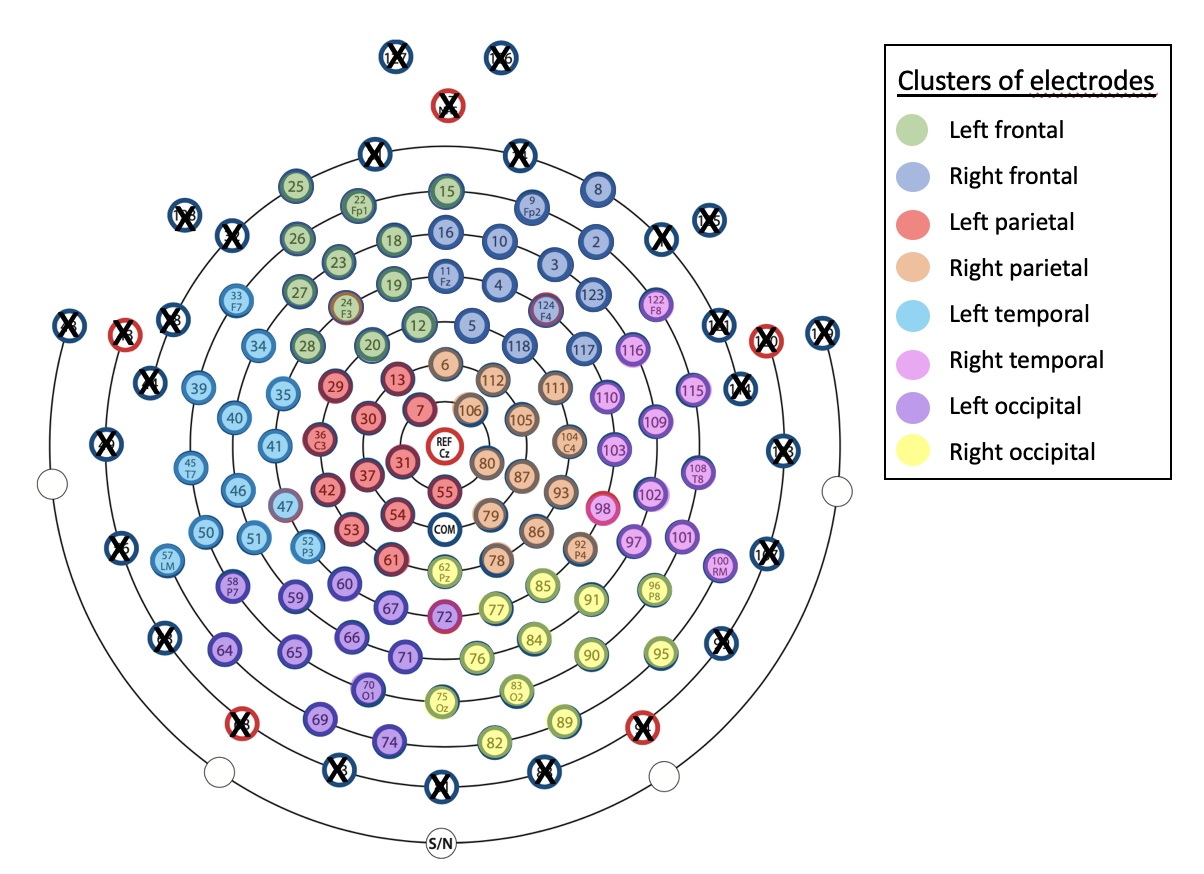


**Figure S1.** Electrode map. Colors represent the clusters of electrodes for comparison of MATLAB and Python preprocessing methods. Electrodes marked with an X (E1, E14, E17, E21, E32, E38, E43, E44, E48, E49, E56, E63, E68, E73, E81, E88, E94, E99, E107, E113, E114, E119, E120, E121, E125, E126, E127, E128) were excluded as they are sensible to muscular artifacts.


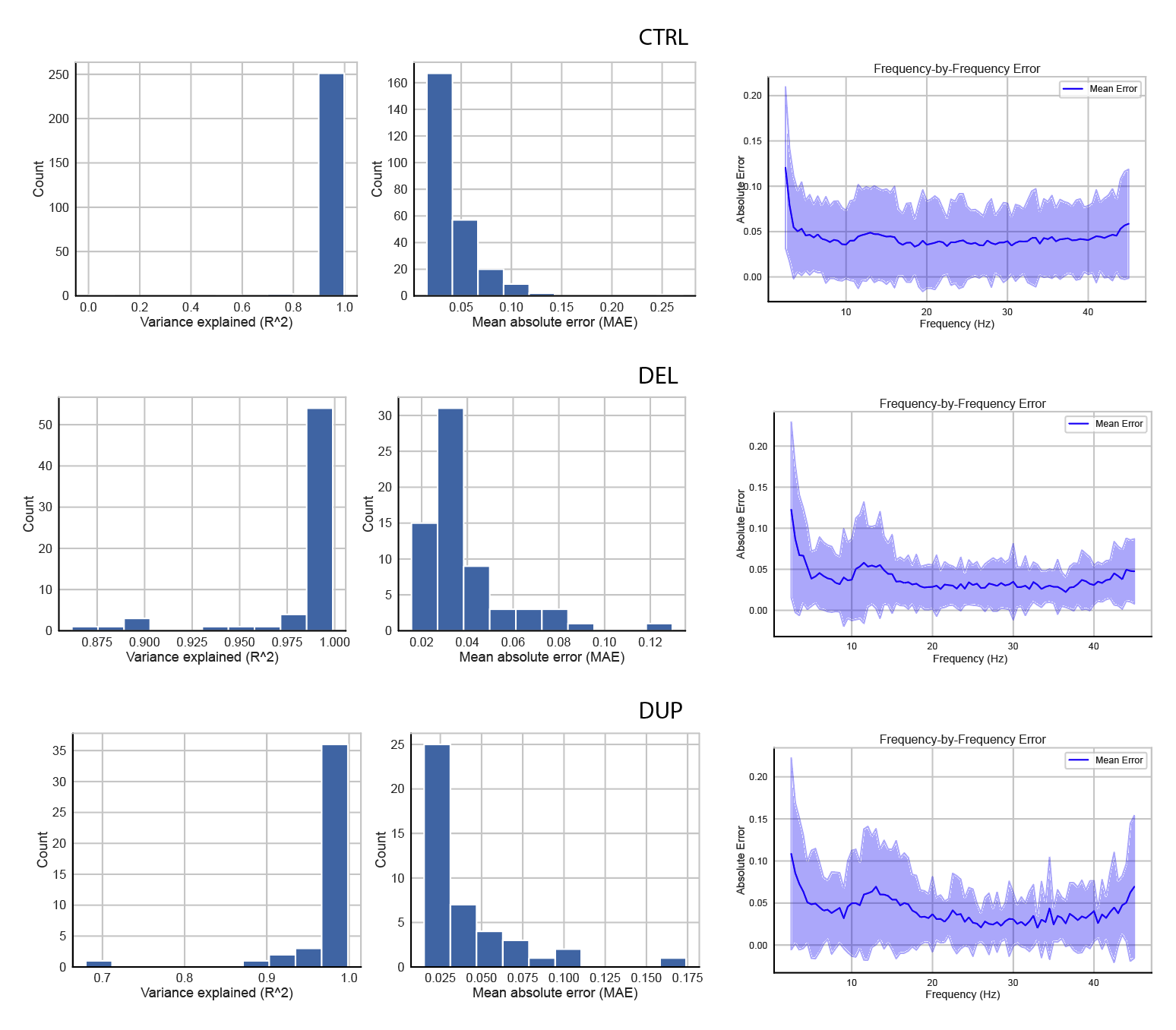


**Figure S2.** (A) Histograms for variance explained (R^2) and mean absolute error (MAE) for DEL, DUP and CTRL. (B) Mean error per frequency with standard deviation in error per frequency (blue shading)


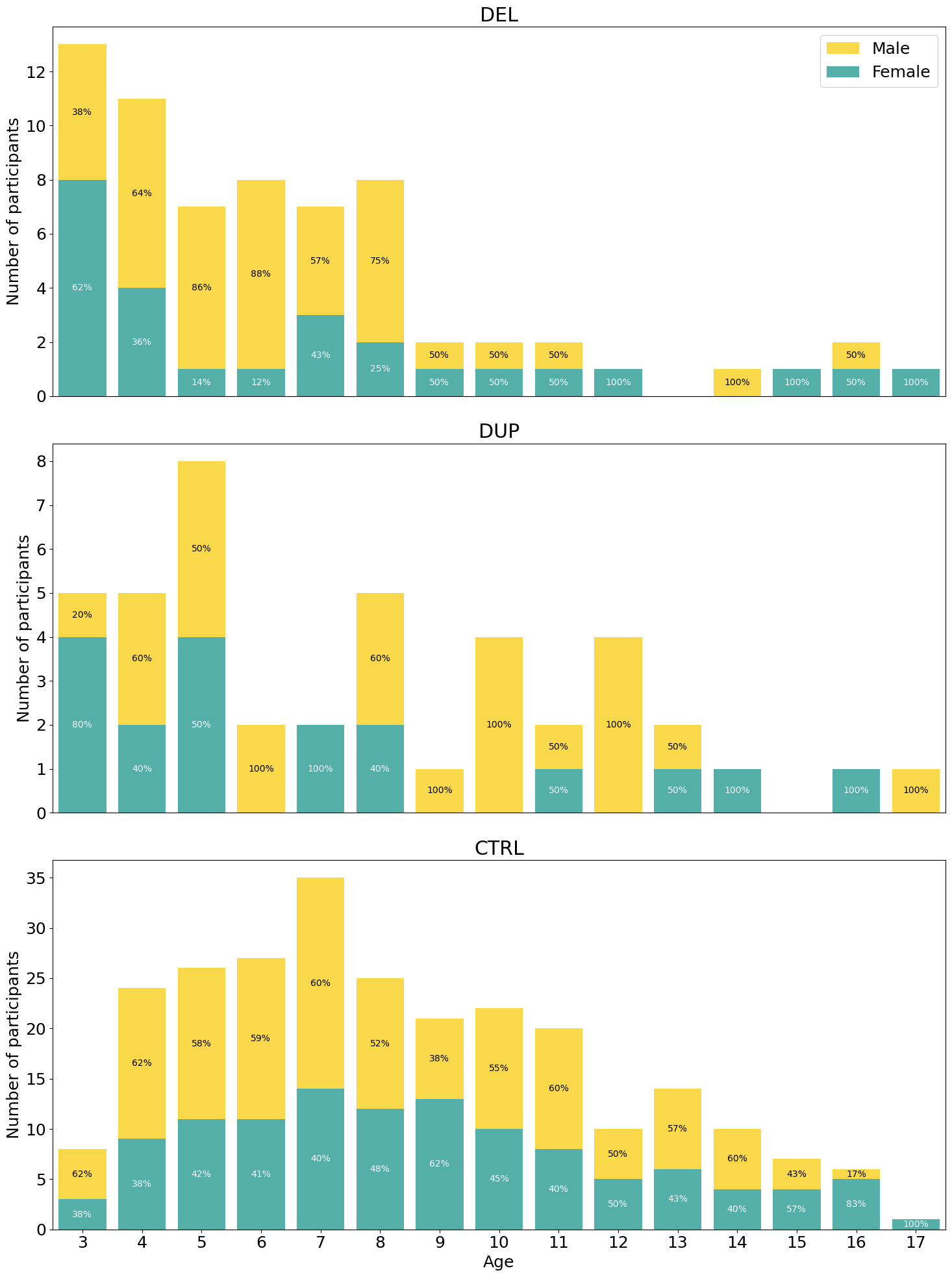


**Figure S3.** Histograms for sex distribution of participants by age for CNV carriers (DEL and DUP) and neurotypical controls (CTRL).


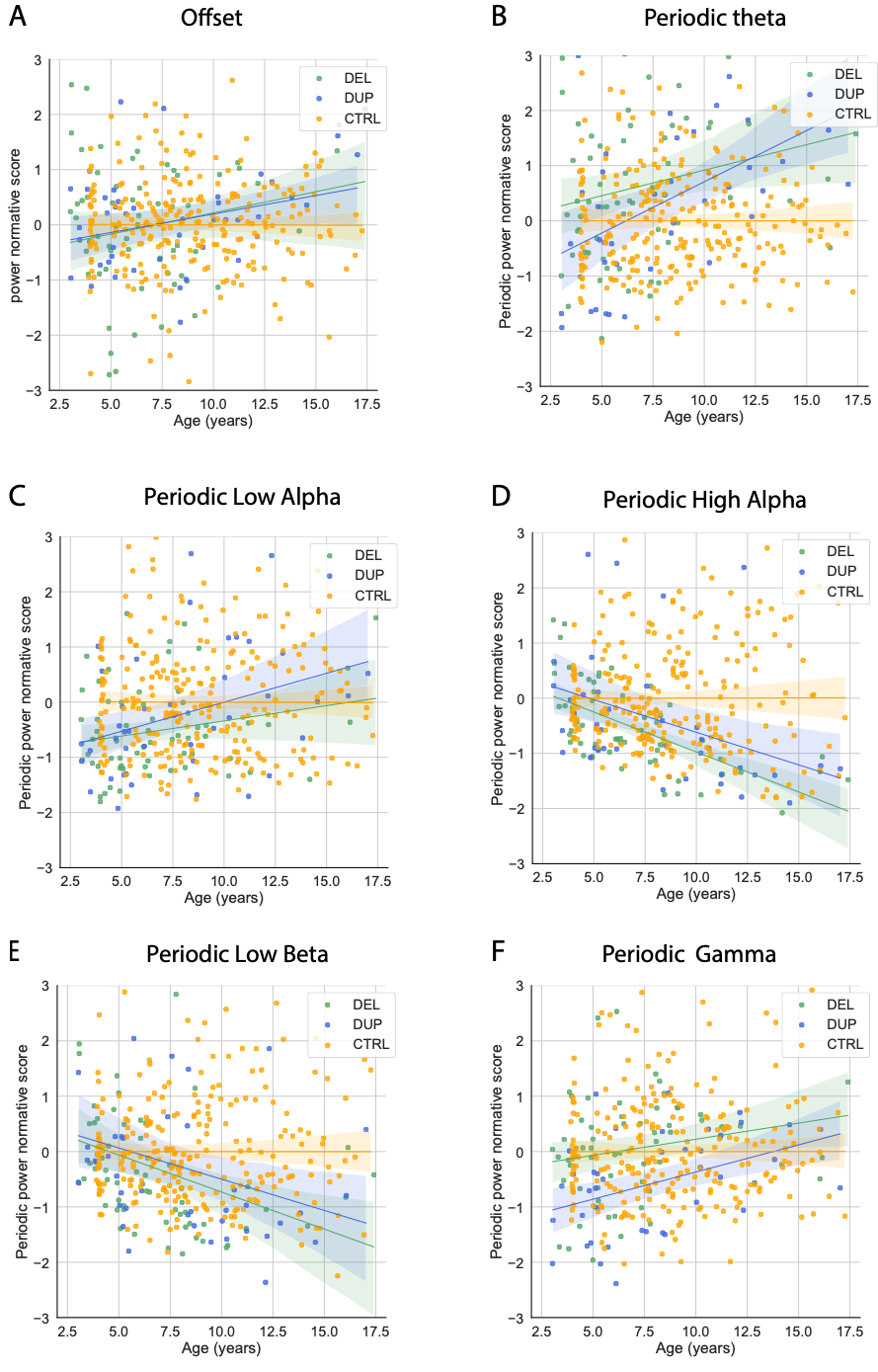


**Figure S4**. Age-normative trajectories in CNV carriers (DEL and DUP) and neurotypical controls (CTR) for significant interaction terms. (A) Offset. (B) Periodic Theta. (C) Periodic Low Alpha. (D) Periodic High Alpha. (E) Periodic Low Beta. (F) Periodic Gamma.


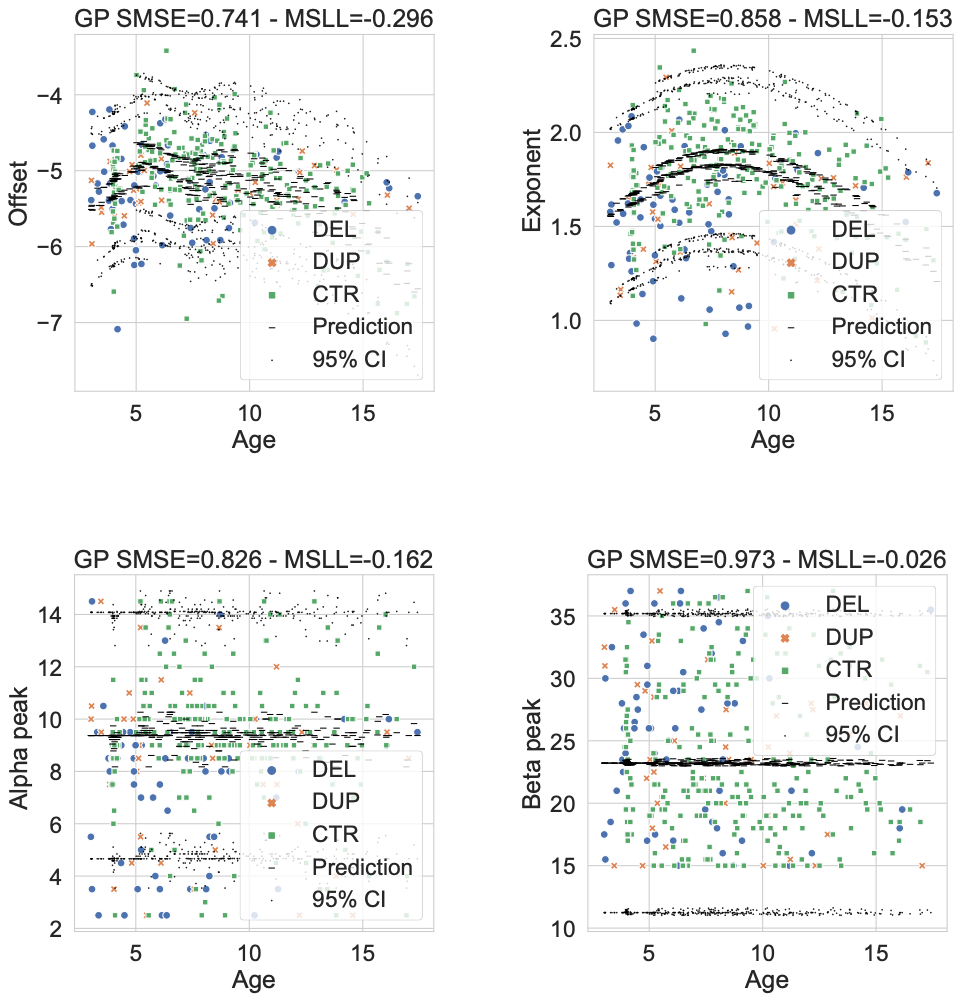


**Figure S5.** Typical developmental trajectory of the aperiodic components (top) and the frequency peaks (bottom) observed in the control group.


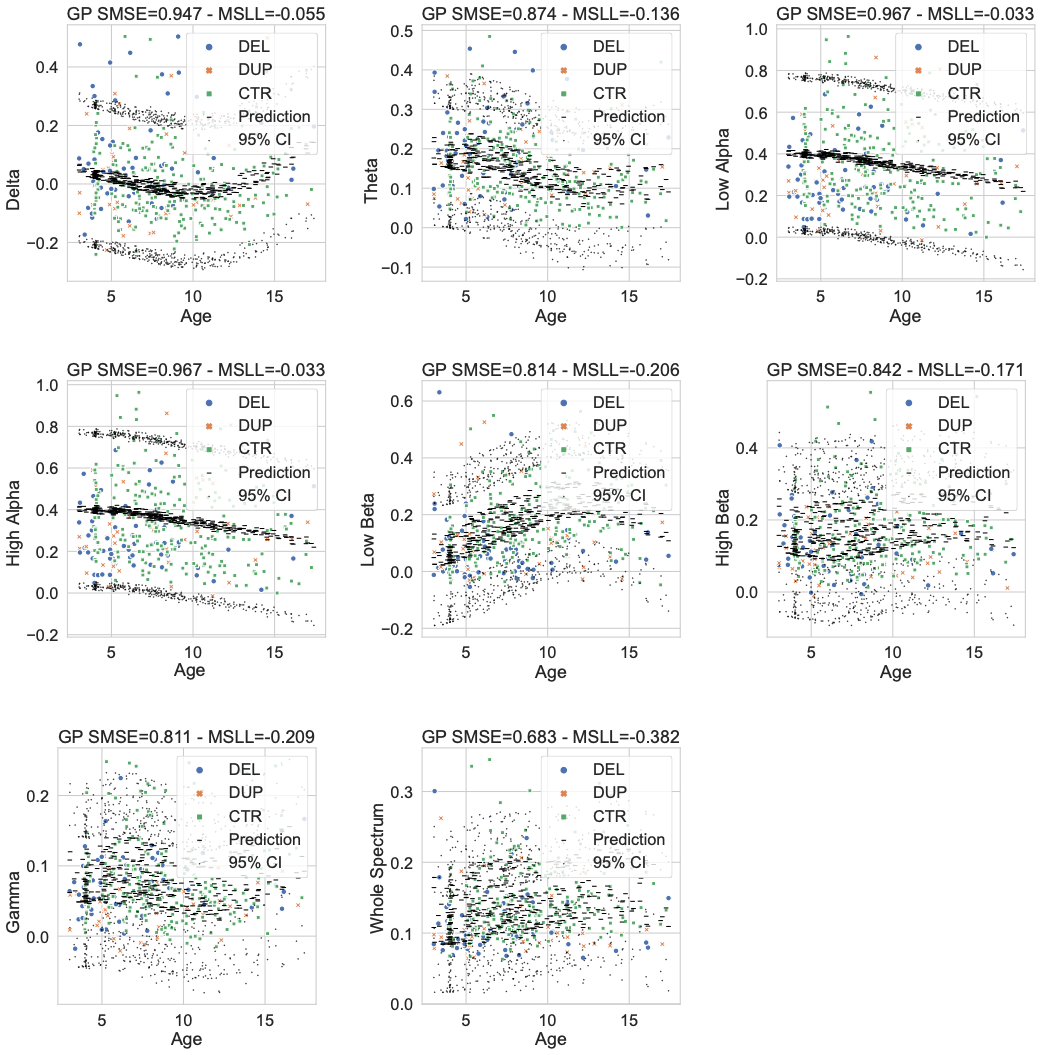


**Figure S6.** Typical developmental trajectory of the periodic signal observed in the control group.


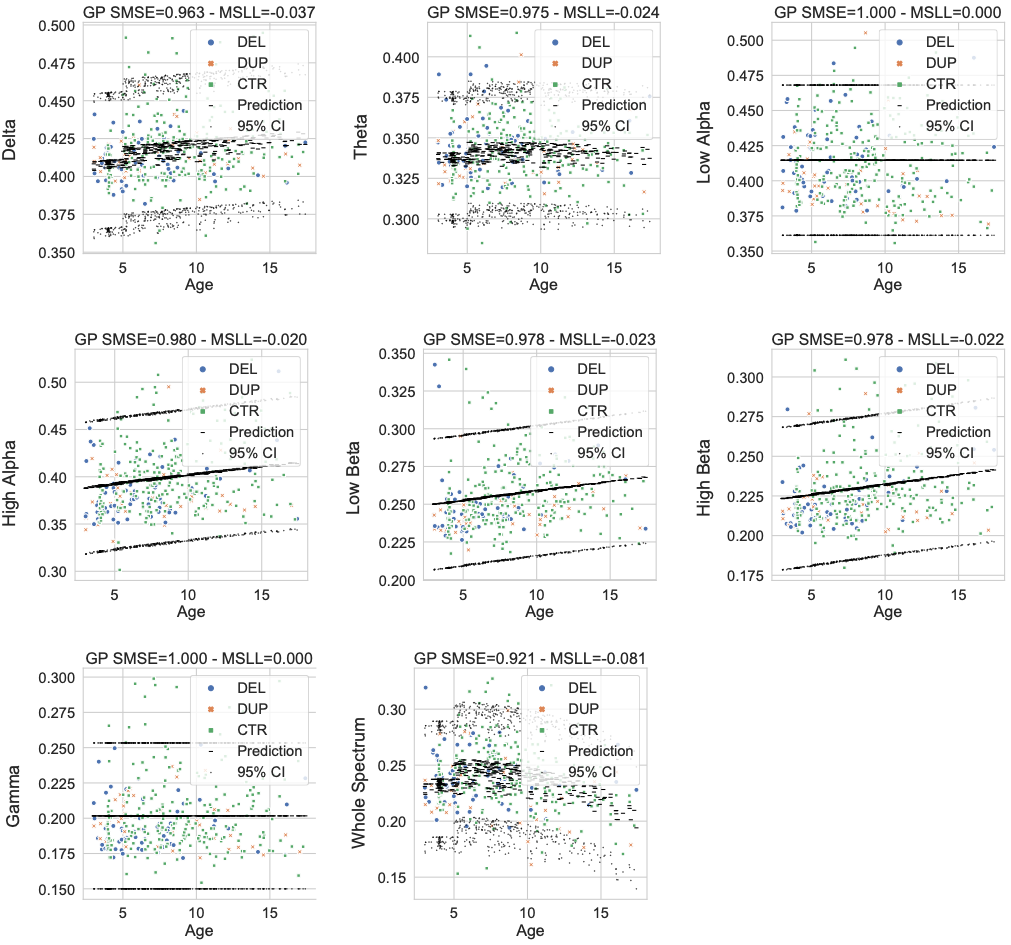


**Figure S7.** Typical developmental trajectory of the connectivity observed in the control group.

**Table S1.** Participants’ CNVs organized according to the chromosome affected. (A) is for deletions and (B) is for duplications.


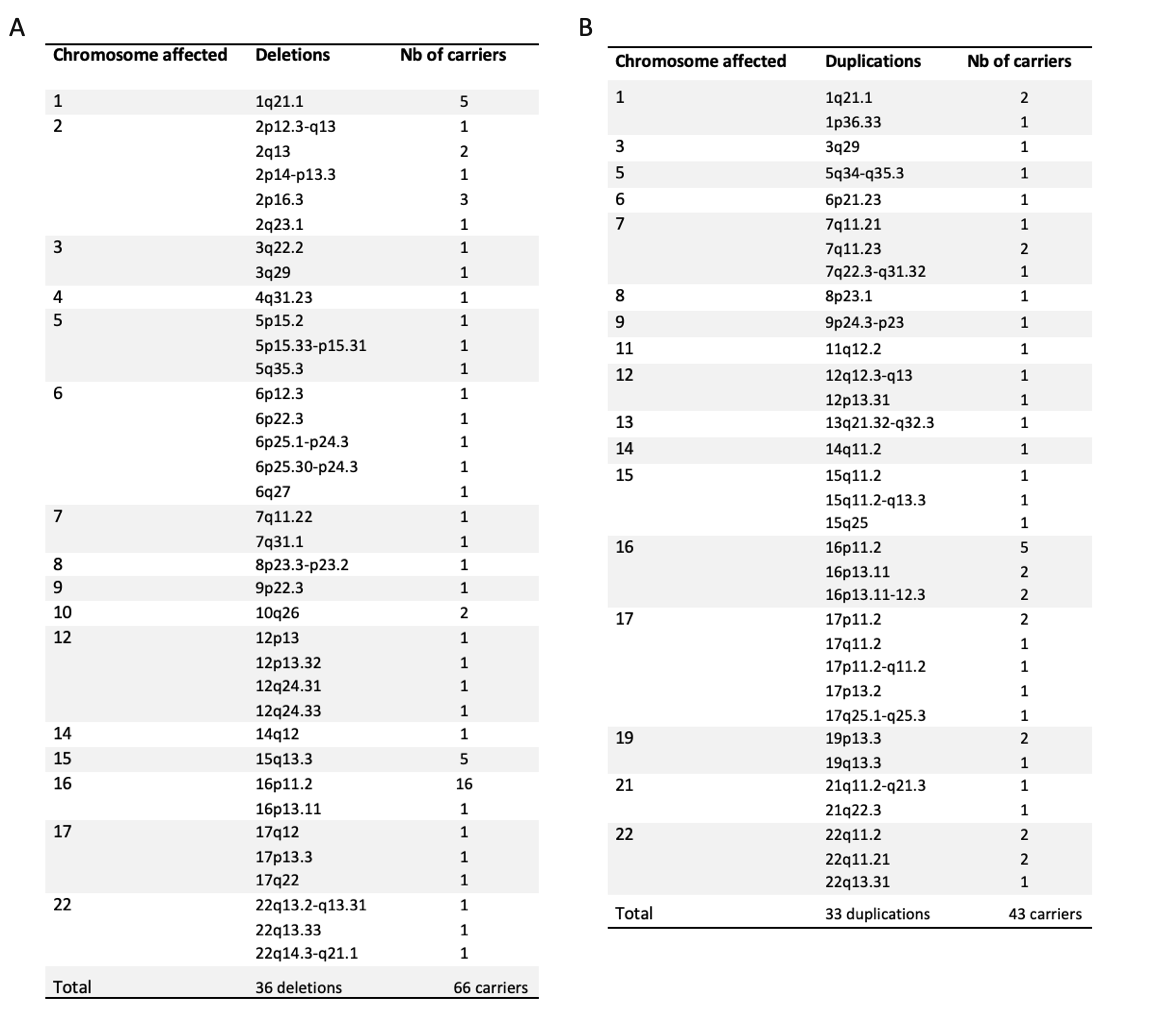


**Table S2**. Regression models of aperiodic signal (z-scores) in CNV carriers (DEL and DUP) and neurotypical controls.

**
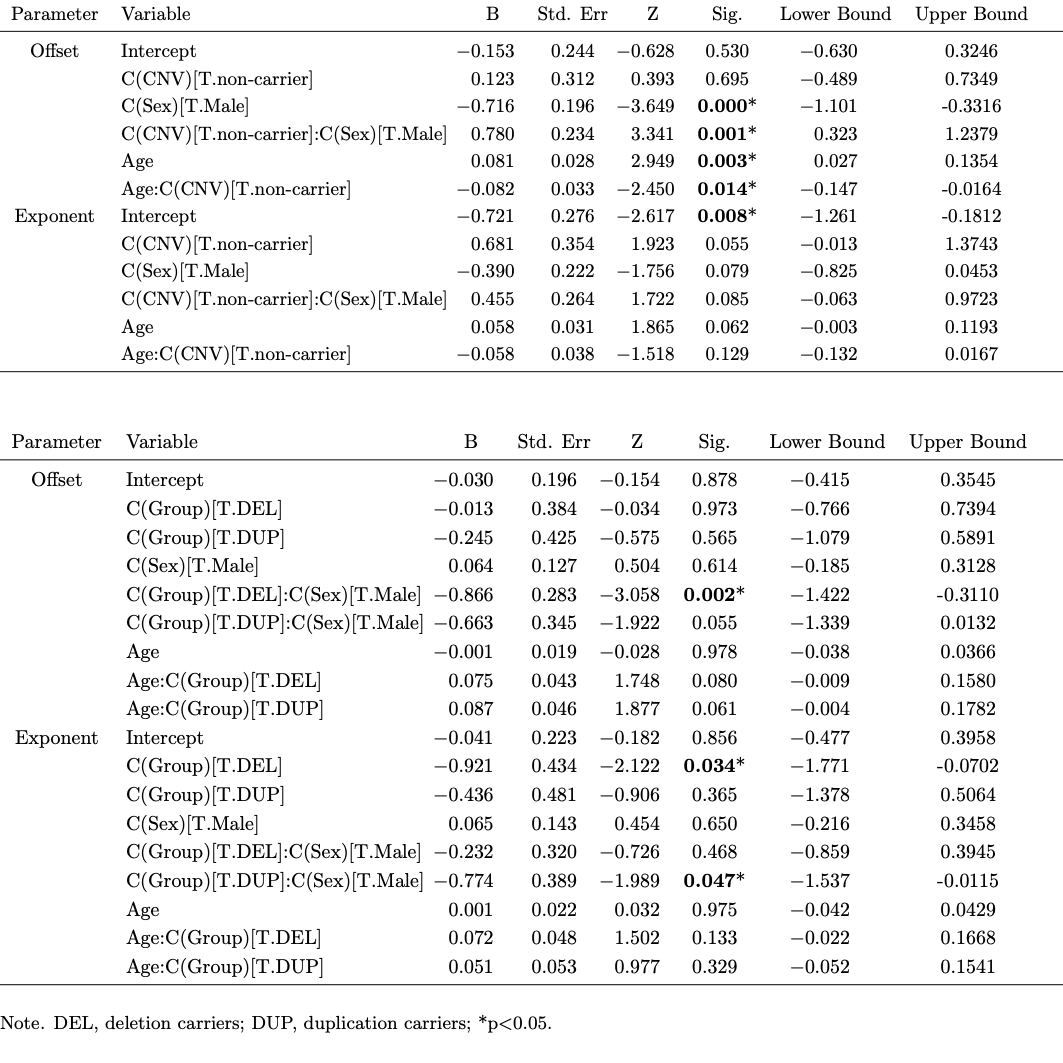
**

**Table S3**. Regression models of periodic signal (z-scores) in CNV carriers (DEL and DUP) and neurotypical controls.

**
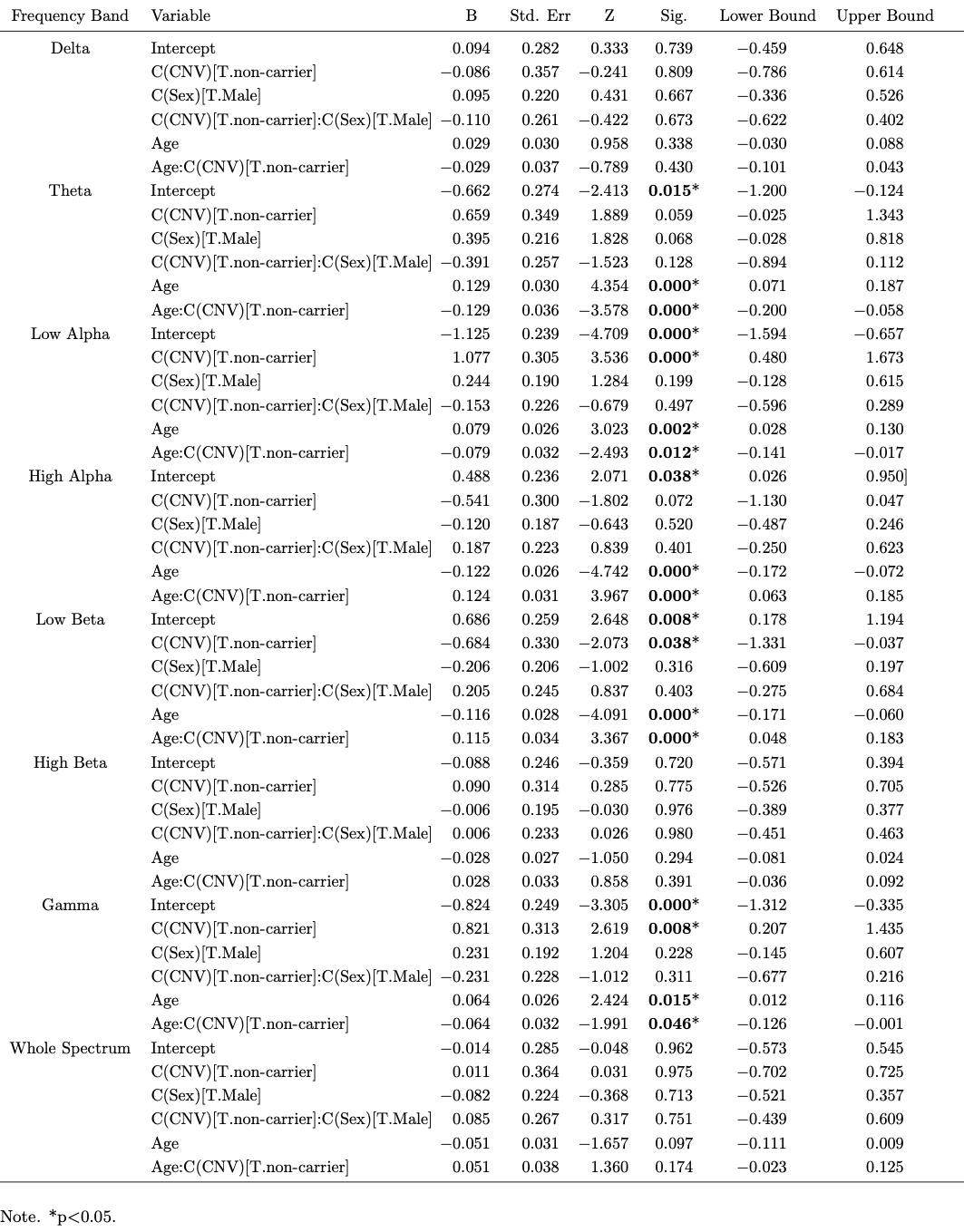
**

**
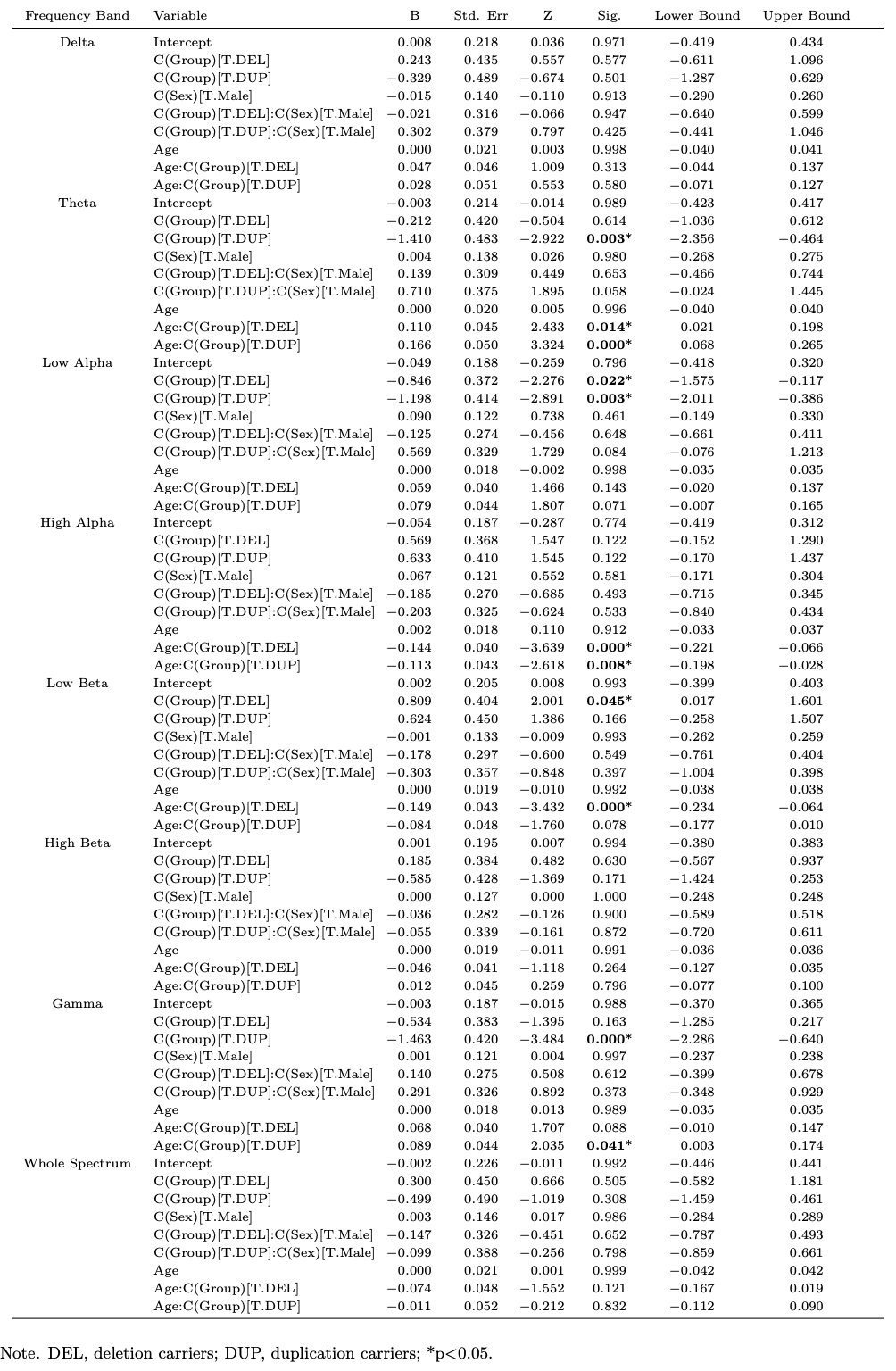
**

**Table S4**. Regression models of frequency peaks (z-sores) in CNV carriers (DEL and DUP) and neurotypical controls.


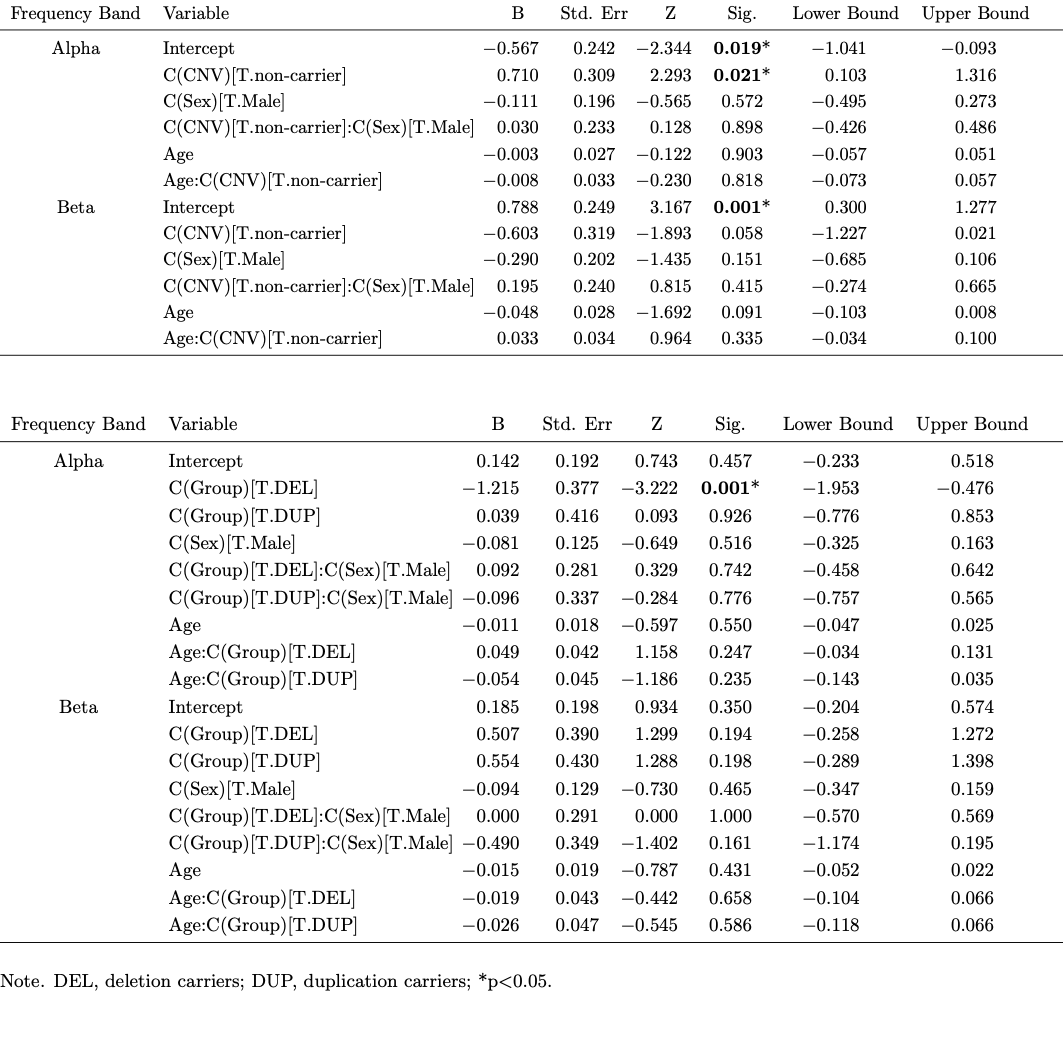


**Table S5**. Regression models of connectivity (z-scores) in CNV carriers (DEL and DUP) and neurotypical controls.

**
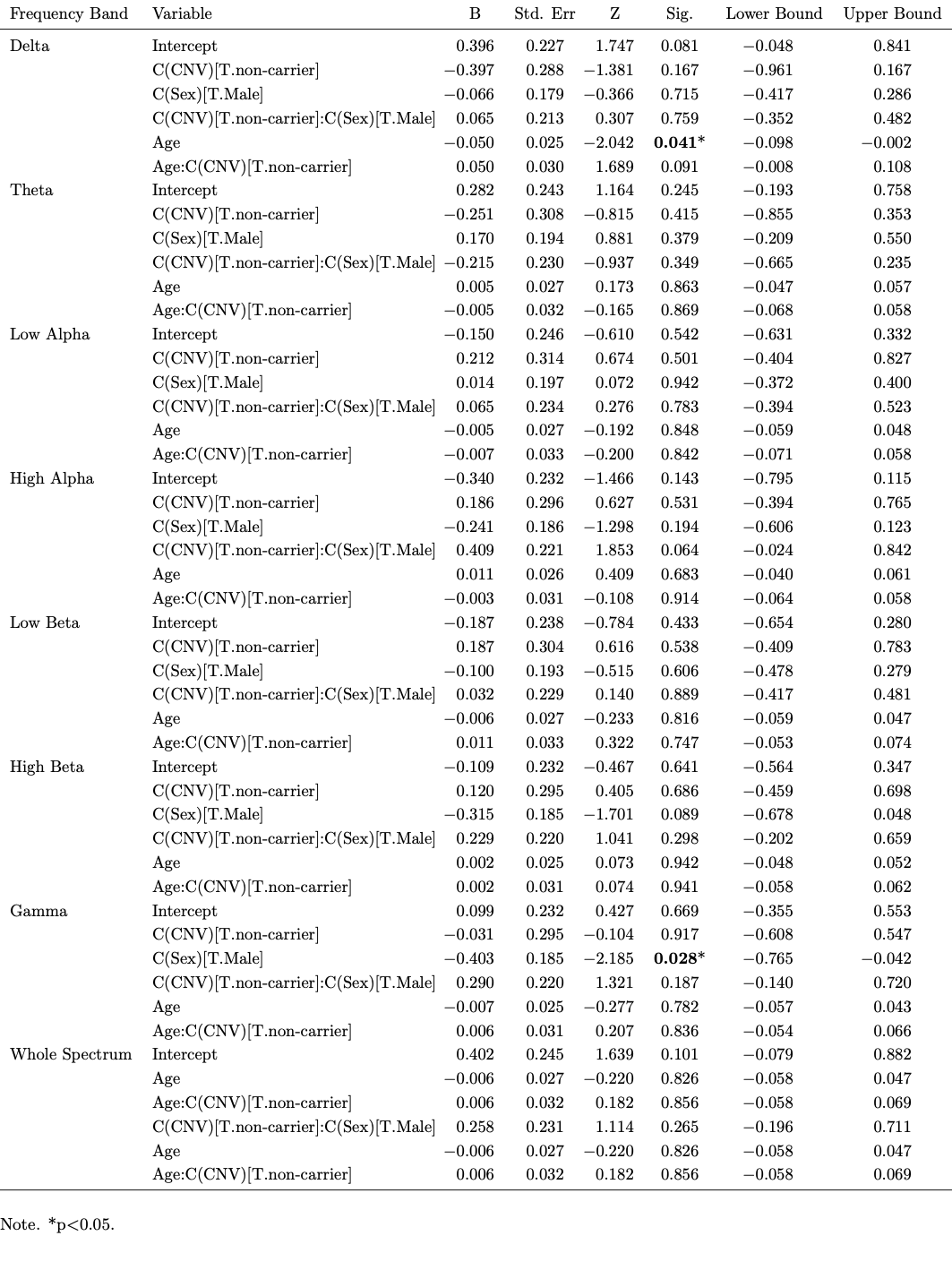
**


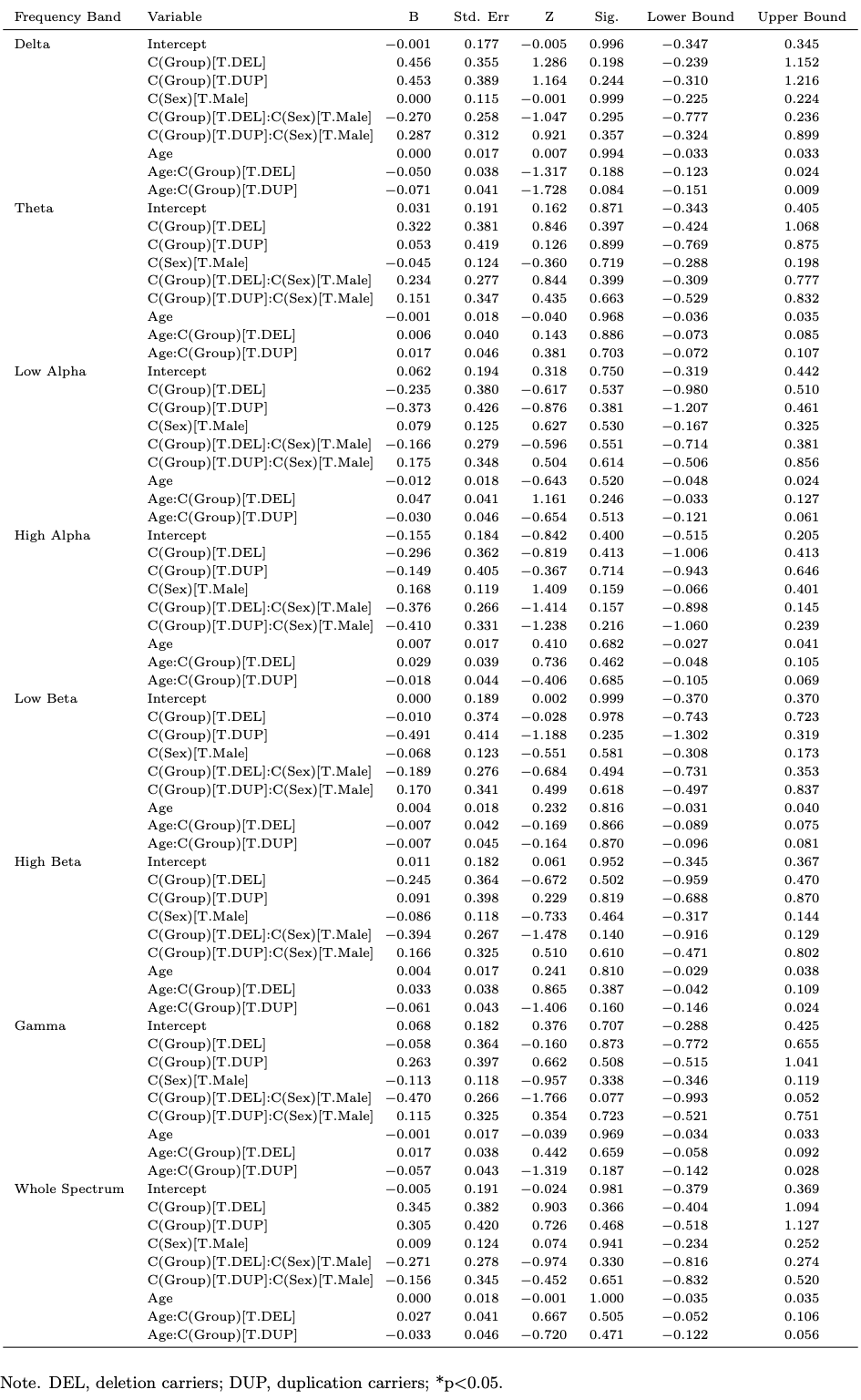

Supplement: Supplementary file 1 — Supplementary material [file 41398_2025_3324_MOESM1_ESM.docx]
